# Human vagus nerve fascicular anatomy and its implications for targeted cardiac stimulation: a microCT segmentation and histological pilot anatomical study

**DOI:** 10.3389/fnins.2026.1731234

**Published:** 2026-02-18

**Authors:** Nicole Thompson, Svetlana Mastitskaya, Francesco Iacoviello, Felicia Turhani, Paul R. Shearing, Kirill Aristovich, David Holder

**Affiliations:** 1 Department of Medical Physics and Biomedical Engineering, University College London, London, United Kingdom; 2 Electrochemical Innovations Lab, Department of Chemical Engineering, University College London, London, United Kingdom

**Keywords:** fascicles, fascicular anatomy, histology, human vagus nerve, microCT, neuroanatomy, neurophysiology, organotopic organization

## Abstract

**Introduction:**

The functional anatomy of autonomic nerve fascicles has remained poorly understood. Building on prior evidence of organotopic organization in the pig cervical vagus nerve, this study examined the thoracic branches of the human vagus nerve using microcomputed tomography (microCT) and histological validation.

**Methods:**

Left and right vagus nerves (*n* = 10) were dissected from human cadavers with cardiac, recurrent laryngeal, and pulmonary branches preserved. Fascicles were segmented and traced within 5 nerves from their branching points, and morphological features analyzed.

**Results:**

Cardiac, pulmonary, and recurrent laryngeal fascicles preserved partial organization near their entry points but merged further along the nerve. In left nerves, cardiac and pulmonary fascicles merged while recurrent laryngeal fascicles remained separate; in right nerves, cardiac fascicles merged with both pulmonary and recurrent laryngeal fascicles. Right nerves had a larger diameter and contained more fascicles, with counts varying along their length, indicative of the observed anastomoses.

**Discussion:**

Notably, the superior cardiac branch on both sides remained distinct near the typical vagus nerve stimulation cuff site, highlighting potential for targeted cardiac neuromodulation potentially relevant to conditions including myocardial infarction, heart failure, and atrial fibrillation. These findings advance understanding of human vagus nerve organization and support the design of selective stimulation strategies for precise autonomic regulation.

## 1 Introduction

The functional anatomy of somatic peripheral nerves has been extensively studied using histological tracing, which has revealed a logical mapping of fascicles to dermatomes and muscle groups (Bäumer et al., 2015; Shearer, 2019). However, the anatomical relationship and functional organization of fascicles within the human vagus nerve, the main peripheral nerve of the autonomic nervous system (ANS) that innervates numerous visceral organs in the thorax and abdomen, as well as the larynx (Thompson et al., 2019), remains largely unknown (Pelot et al., 2020; Ravagli et al., 2020; Rea, 2014). Understanding the organization of fascicles in the vagus nerve could underpin studies on neural control, neurophysiology, and clinical applications such as nerve repair and vagus nerve stimulation (VNS) (Ardell et al., 2015; Bai et al., 2019; Capllonch-Juan and Sepulveda, 2020; Isabella et al., 2021; Mastitskaya et al., 2021; Plachta et al., 2014; Rajendran et al., 2016; Rajendran et al., 2019; Sheheitli and Jirsa, 2020; Thompson et al., 2019).

It may be possible to modulate specific target organs or functions with the use of spatially selective vagus nerve cuffs. This could help avoid off-target effects commonly observed, such as cough, dyspnea, and bradycardia (Fitchett et al., 2021; Mulders et al., 2015). This requires an understanding of the fascicular organization within the vagus nerve, at the level of cuff placement in the upper neck. To date, only four histological studies have examined cross-sections of human vagus nerves at the cervical level, with neither data available on the rest of the nerve nor comprehensive tracing of fascicles along its length (Bäumer et al., 2015; Pelot et al., 2020; Shearer, 2019; Thompson et al., 2019).

Using fascicular histological tracing with microCT, selective stimulation and fast-neural electrical impedance tomography (fnEIT), it was found that there are fascicles in the cervical vagus nerve of the pig which are specific to cardiac, laryngeal or pulmonary function. This suggests that fascicles in the ANS are organized, to a significant extent, according to the function or organ controlled. If a similar organization is found in humans, it could lead to a paradigm shift in vagus nerve stimulation, allowing selective, targeted stimulation. This could not only advance its current clinical applications in epilepsy and depression by improving therapeutic efficacy by avoiding off-target effects (Bonaz et al., 2019; Breit et al., 2019; Browning et al., 2017; Koopman et al., 2016) but also allow for the extension of VNS to other disorders such as heart failure.

In this study, the purpose was to trace the cranial representation of the cardiac, pulmonary, and recurrent laryngeal thoracic branches in cadaver human vagus nerves (*n* = 5, 3 left, 2 right vagi) using microCT. Additionally, we performed histology and immunohistochemistry (IHC) at regular intervals along the vagal trunk and from each nerve branch (*n* = 10, 5 left, 5 right). We aimed to address the following questions:

1. Is there organotopic organization in the fascicles of the human cervical vagus nerve, similar to the pig model?

2. How do specific histological and immunohistochemical markers reveal functional or anatomical differences in the fascicles of the human cervical vagus nerve?

## 2 Materials and methods

### 2.1 Experimental design

Previously, Neurolucida 360 was used to manually trace the fascicles of the vagus nerve. However, Vesselucida 360, despite being designed for vessels, provided a more seamless and less time-consuming tool for tracing and segmenting fascicles of the vagus nerve from their branching point till cervical level. This was the only software used in this study for tracing fascicles.

Despite only having fascicular, and not fiber, resolution of microCT as mentioned above, by performing this microCT and tracing method, the following can be determined, quantified, analyzed, and subsequently compared between individuals:

1. the trajectory of fascicles within the nerve from entry into the vagus nerve to the cervical level;
2. the spatial arrangement of fascicles at the cervical level;
3. the longitudinal course and reorganization of fascicles along the nerve;
4. the number of fascicles within the vagus nerve and within each branch;
5. the number of branches and the distances between branches to specific organs and effectors (gross vagal anatomy);
6. the distances between the first and last merging events of fascicles associated with specific organ targets within the vagus nerve, reflecting the length over which fascicles travel as discrete cable-like structures prior to merging with other fascicle populations (fascicular morphology);
7. the frequency of merging and splitting events between fascicles of the same type and of different types, including the degree of fascicular crossover and plexiform organization of the nerve.

Routine histology and immunohistochemistry (IHC) are useful for identifying nerve anatomical features like nerve diameter, fascicle count and diameter, fiber count, and fiber diameter/type. IHC can further characterize nerves based on neurochemical/microtubule characteristics, distinguishing different fiber types. Trichrome staining uses 3 dyes to stain collagen, keratin, muscle fibers, cytoplasm, nuclei, and bone (O’Connor and Valle, 1982). By immunostaining with neurofilament (NF) and myelin basic protein (MBP) antibodies on one slide, accurate detection of nerve fiber sizes is possible, visualizing intermediate filaments (Yuan et al., 2012) and myelin sheaths (Deber and Reynolds, 1991) and thereby identifying both myelinated and non-myelinated fibers.

Due to limitations imposed during the Covid pandemic, timeframes with the human vagus nerve samples and entry rules into labs not within our department, some aspects of this study were affected. The original target was to perform both histology, including Trichrome and H&E, and IHC, including ChAT, and NF and MBP, on all sections from the ten nerves. Due to the timing and practical constraints, all except ChAT could be performed on the sections. ChAT could not be optimized within this timeframe for the use on human tissue and thus, was subsequently removed from the study. Although the remaining staining techniques were performed, if a cross section of nerve was missing from the section, this could not be corrected for prior to nerve tissue being returned to the facility. This work still, however, serves as a preliminary tracing and staining study for work to be done in the future. Ten vagus nerves (five left and five right) were successfully microCT scanned and reconstructed. Only three left and two right vagus nerves which have been fully traced from caudal to cranial level are presented in this manuscript.

### 2.2 Human vagus nerve samples

The Evelyn Cambridge Surgical Training Centre, A Cambridge University Health Partners Facility, loaned human vagus nerve samples for this study. The Human Tissue Authority (HTA) Designated Individual (DI), namely Dr. C R Constant, approved the loan of human tissue from first-person, signed and witnessed consented (willed) donors for the use in this project. Dissection of vagus nerves from the donors was supervised by the HTA DI for the Centre’s license and all subsequent transport and work on the nerves was pre-approved by the HTA DI on their license and specified in a Material Transfer/Loan Agreement. Separate ethical approval was not required. Further information on the cadavers can be found in Supplementary Table 1.

### 2.3 Post-mortem dissection and sample collection

Both left and right vagus nerves from 5 frozen human cadavers were dissected from the cervical region down to the branching regions of cardiac, recurrent laryngeal and pulmonary branches; all the branches left attached to the main trunk of the vagus nerve. The nerves were exposed using techniques that are similar to surgery for vagus nerve electrode placement. A linear incision through the skin and platysma muscle was made one centimeter above the clavicle extending cranially and parallel to the anterior border of the sternocleidomastoid muscle. This muscle was retracted laterally to expose the carotid sheath. The omohyoid muscle was then dissected from the carotid sheath and retracted cranially. The carotid sheath was then opened, and the vagus nerve was exposed between the common carotid artery and jugular vein. At this level (5 cm caudal from nodose ganglion), the vagus nerve was transected and exposed to follow it caudally toward any and all branches leading to the heart, lungs and larynx including the superior and inferior cardiac branches, pulmonary branches and the recurrent laryngeal branch. The vagus nerve was resected from below the last pulmonary branch to 2 cm cranial to the average VNS cuff placement and 5 cm caudal from the nodose ganglion, yielding samples about 25 cm in length. Sutures were placed around the vagus nerve prior to any branching region (i.e., the region where a branch leaves the main vagal trunk) to mark the branching point. Nerves were then placed in neutral buffered formalin (10%) for fixation, labeled accordingly and transported to UCL. All ten nerves were iodine stained, scanned by microCT, and had histology and IHC performed; five nerves proceeded to computerized segmentation to identify fascicle anatomy.

### 2.4 Pre-processing and staining

Pre-processing and staining were performed in the same way as the pig vagus nerves in Thompson et al. (2023). After fixation, nerve samples were measured, sutures of 1 cm length were superglued to the vagal trunk in 4 cm intervals, and nerves cut into 4 cm lengths at the level of suture placement leaving half of the suture on the end of each section as a marker for subsequent co-registration. Two to three sections were placed into a tube of 50 mL Lugol’s solution (total iodine 1%; 0.74% KI, 0.37% I) (Sigma Aldrich L6141) for 5 days (120 h) prior to scanning to achieve maximum contrast between fascicles and the rest of the nerve tissue. On the day of the microCT scan, the nerve was removed from the tube and blotted dry on paper towel to remove any excess Lugol’s solution. The nerve sections were placed next to each other onto a piece of cling film (10 × 5 cm) (Tesco, United Kingdom) in order from cranial to caudal along the length of the nerve, with cranial ends at the top, and sealed with another piece of cling film to retain moisture during the scan as to avoid shrinkage of the nerve tissue. The sealed nerve samples were rolled around a cylinder of sponge (0.5 cm D × 4.5 cm) and wrapped in another two layers of cling film to form a tightly wound cylinder with a diameter of ∼1.5 cm to fit within the field of view at the required resolution. The wrapped cylinder was placed within a ∼2 cm sponge inside a 3D-printed mount (30 mm width × 60 mm height tube in a mount fitted to the Nikon XT H 225 scanner stage; stl files available in Supplementary files) filled with ∼2 cm layer of sponge around the edges, ensuring a tight fit and the ends sealed with tape (Transpore, 3M, United Kingdom).

### 2.5 MicroCT scanning and reconstruction

A microCT scanner (Nikon XT H 225, Nikon Metrology, Tring, United Kingdom) was homed and then conditioned at 200 kVp for 10 min before scanning and the target changed to molybdenum. The scanner was calibrated as per Nikon XT H 225 protocols prior to scanning the nerves. In addition, correction images were obtained prior to each scan for normalization with the detector and any background noise during reconstruction. All nerves were scanned with the following scanning parameters: 35 kVp energy, 120 μA current, 7 W power, an exposure of 0.25 fps, optimized projections, and a resolution with isotropic voxel size of 7 μm. Scans were reconstructed in CT Pro 3D (Nikon’s software for reconstructing CT data generated by Nikon Metrology, Tring, United Kingdom). The center of rotation was calculated manually with dual slice selection. Beam hardening correction was performed with a preset of 2 and coefficient of 0.0. The reconstructions were saved as 16-bit volumes and triple TIFF 16-bit image stack files allowing for subsequent image analysis and segmentation in various software.

### 2.6 Image analysis, segmentation and tracing

Image analysis, segmentation, and tracing were performed on reconstructed microCT datasets from five human vagus nerves (three left and two right). Reconstructed microCT scan images were analyzed in ImageJ (Schindelin et al., 2012) in the XY plane to view the cross-section of the nerve. The vertical alignment of the nerve was positioned so that the cross-sectional plane was viewed in the XY stack and the longitudinal plane in the XZ and YZ stacks. This allowed for validation of the scanning protocol, direction of the nerve, and visual analysis of the quality of the image and the distinguishability of the soft tissues—specifically the identification of the fascicles known to exist within the nerve. AVI files were created from ImageJ to enable stack slice evaluation, identification of suture positions and branching locations of the vagus nerve, and as a reference during segmentation. Image stacks (XY plane along the *Z*-axis) were loaded into Vesselucida 360 (Version 2021.1.3, MBF Bioscience LLC, Williston, VT, United States) and image histograms adjusted to optimize visualization of the fascicles when required. Fascicles of the three target organs/functions (namely, cardiac, recurrent laryngeal and pulmonary fascicular groups) were segmented from the rest of the nerve using Vessel mode from the Trace tools. Starting from identification within branches of the vagus nerve, the fascicles were traced through every slice of each scan proceeding cranially up the length of the nerve to the cervical region at the level of cuff placement. Points adding to the path of the vessel trace were place every 50–100 sections and the thickness of the vessel trace was altered to match the diameter of the fascicle being traced throughout its length. If fascicles merged with or split into others at a higher frequency, points of the vessel trace were placed at smaller intervals to ensure accurate tracing. The vessel trace was labeled according to the organ from which the fascicle originated. If the fascicle split into two or more fascicles, a bifurcating or trifurcating node was inserted and each branch exiting the node traced cranially up the nerve until either the fascicle merged with another fascicle, or another node needed to be created for splitting fascicles. If fascicles merged into one another, the vessel trace was ended and another trace started that was labeled as containing the respective merged fascicle types and therefore containing fibers from different target organs (i.e., pulmonary and recurrent laryngeal merged fascicle). To continue tracing across cut regions of the nerve, the superglued suture markers and distinct physiological regions or landmarks were used to align the cranial and distal ends of the cut nerves and tracing continued (see Supplementary Figure 1 for a visual depiction of how co-registration was performed). Tracing across the cut regions was successful for all nerves. Subsequent to tracing throughout the segments of nerve in each scan, the stack was viewed in the 3D Environment where the traces are visualized in 3D displaying the thickness, path and color (specified when labeling the fascicle traces) of the fascicles throughout the length of each scan. Due to software data size limitations, the overlapping, consecutive scans could not be physically stitched together to show the full length at once, but rather allowing each scan to be visualized separately.

### 2.7 Histology and IHC for morphology analysis and microCT segmentation validation

Subsequent to scanning, stained nerves were placed back into neutral buffered formalin for at least a week which allowed for the Lugol’s solution to be soaked out. The formalin was refreshed weekly prior to histology. Histology and immunohistochemistry (IHC) were performed on all 10 nerves. From each human vagus nerve, 0.5-cm-long segments were excised at predefined anatomical locations: at the level of cuff placement, 2 cm distal to the cuff, and at 4-cm intervals thereafter along the nerve trunk. Additional segments were collected immediately cranial to major branching points and from 0.5 to 1 cm within each branch. This sampling strategy yielded 6–7 segments from the nerve trunk at regular intervals, 3–4 segments cranial to branching regions, and 3–5 segments from the branches, resulting in 9–11 trunk segments and a total of 12–16 segments per nerve for histological and IHC analysis. Segments from the main vagal trunk were placed into a cassette with the order and placement of the segments noted. In another cassette, the segments prior to branching region were placed in order in one row, and those from the branches were placed in order in another row. These nerve samples were embedded in paraffin and six sequential sections cut every 4 μm from each block; two were placed on uncoated slides and three on coated slides. One section of each block on an uncoated slide was stained with Hematoxylin and Eosin (H&E, a routine stain used to demonstrate the general morphology of tissue) (Sheehan and Hrapchak, 1987), the second section on a coated slide stained with Trichrome, and the third section on a coated slide immunostained for both NF (anti-neurofilament heavy polypeptide antibody ab8135 1:1,000) and MBP (anti-myelin basic protein antibody ab7349 1:4,000) with a secondary biotin labeled antibody (donkey anti-rabbit IgG H&L (Biotin) ab6801 1:1,000) (see Supplementary Information). The remaining two sections, one coated and one uncoated, were extra. For H&E staining, a Gemini AS Automated Slide Stainer (Epredia, Portsmouth, New Hampshire, United States) was used. A Leica BOND RX (Leica Biosystems, Milton Keynes, United Kingdom) machine was used for automated IHC. All slides were imaged with light microscopy using a NanaZoomer S360 (Hamamatsu Photonics, Hamamatsu City, Japan).

Identification of histopathological features was performed, and the images of the histology and immunohistochemistry cross-sections were then compared to the corresponding slice in the microCT scan of the same nerve for comparison and validation. The presence and number of fascicles visualized in the golden standard of histology were compared to those identified during segmentation of microCT scans to confirm that the segmentation results correlated (Supplementary Figures 5, 6 in Thompson et al., 2023). In addition, fiber sizes within organ-specific fascicles were observed.

### 2.8 Nerve analysis

NDP.view 2 (Hamamatsu Photonics, Hamamatsu City, Japan) was used to view the images of the histology and IHC cross-sections on the multiple slides. Using NDP.view 2 annotation tools, namely “ruler” and “freehand region,” nerve dimensions were quantified from the H&E slides at the mid-cervical level (Supplementary Tables 3, 4, 6). As the nerves exhibit non-uniform or non-circular cross-sectional morphology, both the short-axis and long-axis diameters were measured; the reported diameter represents the mean of these two values. Cross-sectional area and circumference were measured using the freehand region tool, which allowed precise tracing of the nerve boundary and accounted for irregular nerve geometry. This approach avoided assumptions of circularity and minimized geometric bias that would arise from estimating area or circumference based solely on diameter measurements. These were then averaged for the ten nerves, and for the left and right nerves, respectively.

Intra- and inter-observer repeatability of morphometric measurements was assessed using repeated measurements of a single human nerve cross-section. Three independent researchers each performed nine repeated measurements of nerve circumference, cross-sectional area, short diameter, long diameter, and mean diameter. Researchers were blinded to each other’s measurements during analysis. Intra-observer repeatability was high, as demonstrated by very low within-researcher standard deviations and variance, narrow 95% confidence intervals, and low coefficients of variation across all morphometric parameters (Supplementary Table 2). Inter-observer agreement was assessed using one-way ANOVA followed by Tukey’s corrected pairwise comparisons between researchers. No statistically significant differences were observed between any researcher pairs for any morphometric parameter (all adjusted *p* > 0.05, Supplementary Table 2). Collectively, these findings demonstrate excellent intra- and inter-observer repeatability, indicating that the morphometric measurements are robust, reproducible, and not dependent on the individual researcher. This supports the reliability of all nerve morphometric measurements reported in this study. Fascicle counts was also consistent between researchers.

The number of fascicles (and fascicle bundles—defined as the number of fascicles separated by thin layer of perineurium from but within the same thicker layer of perineurium as other fascicles) were counted at regular intervals along the trunk of the nerve and within the branches for the ten human vagus nerves from the histology images. Specifically, the Trichrome slides were used for this for clear distinguishability of the fascicles from the rest of the nerve tissue. In the cases where a nerve cross-section was not visible on the slides, H&E slides were used. This was compared between nerves (Supplementary Tables 4, 5). The distinguishability of the fascicles and fascicle bundles was visible upon analysis of the microCT scans, when visualizing the movement of the fascicles in the Z direction; however, for unambiguous identification of this, the Trichrome histology slides were used. The distance between branches was calculated for all dissected nerves and averaged to provide general gross vagal anatomical information and compared between individuals (Supplementary Table 6). Additionally, the cervical vagus nerve average diameters, areas and fascicle counts were compared to histological studies (Table 1; Hammer et al., 2015; Hammer et al., 2018; Pelot et al., 2020; Stakenborg et al., 2020; Verlinden et al., 2016).

**TABLE 1:** Cervical human vagus nerve morphology compared between studies.

Study	Methods (to take into consideration for variation in shrinkage)				
	Cadaver state		Nerve fixation		Embedding
Thompson (this study)	Fresh frozen, defrosted		10% Neutral buffered formalin		Paraffin
Pelot et al. (2020)	Embalmed		4% PFA		Paraffin
Hammer et al. (2015) 2018	Ethanol-glycerin protocol		Casted and fixed		Alginic acid
Verlinden et al. (2016)	Formalin-fixed		Post-fixated in OsO4		Paraffin
Stakenborg et al. (2020)	Formalin-fixed		4% PFA		Tissue-Tek OCT compound
**Area (mm^2^)**	**Mean**	**SD**			
Thompson (this study)	3.63	1.26			
Pelot et al. (2020)	3.04	1.40			
Hammer et al. (2015) 2018	7.07	2.95			
Verlinden et al. (2016)	7.72	2.73	**Mean**	**SD**	
Stakenborg et al. (2020)	3.20	4.93	2.27		
**Diameter (mm)**	**Mean**	**SD**			
Thompson (this study)	2.30	0.44			
Pelot et al. (2020)	1.90	0.42			
Hammer et al. (2015) 2018	2.99				
Verlinden et al. (2016)	3.13		**Mean**	**SD**	
Stakenborg et al. (2020)	2.02	0.35	2.47	0.56	
**Fascicle count**	**Mean**	**SD**			
Thompson (this study)	10.60	4.16			
Pelot et al. (2020)	5.90	2.42			
Hammer et al. (2015) 2018	6.70	4.50			
Verlinden et al. (2016)	5.15	3.35			
Stakenborg et al. (2020)	6.50	5.00	**Mean**	**SD**	
Thompson (this study, fascicle bundles)	7.00	3.00	6.25	1.89	

## 3 Results

### 3.1 MicroCT scanning and fascicle tracing of human vagus nerves

**FIGURE 1:**
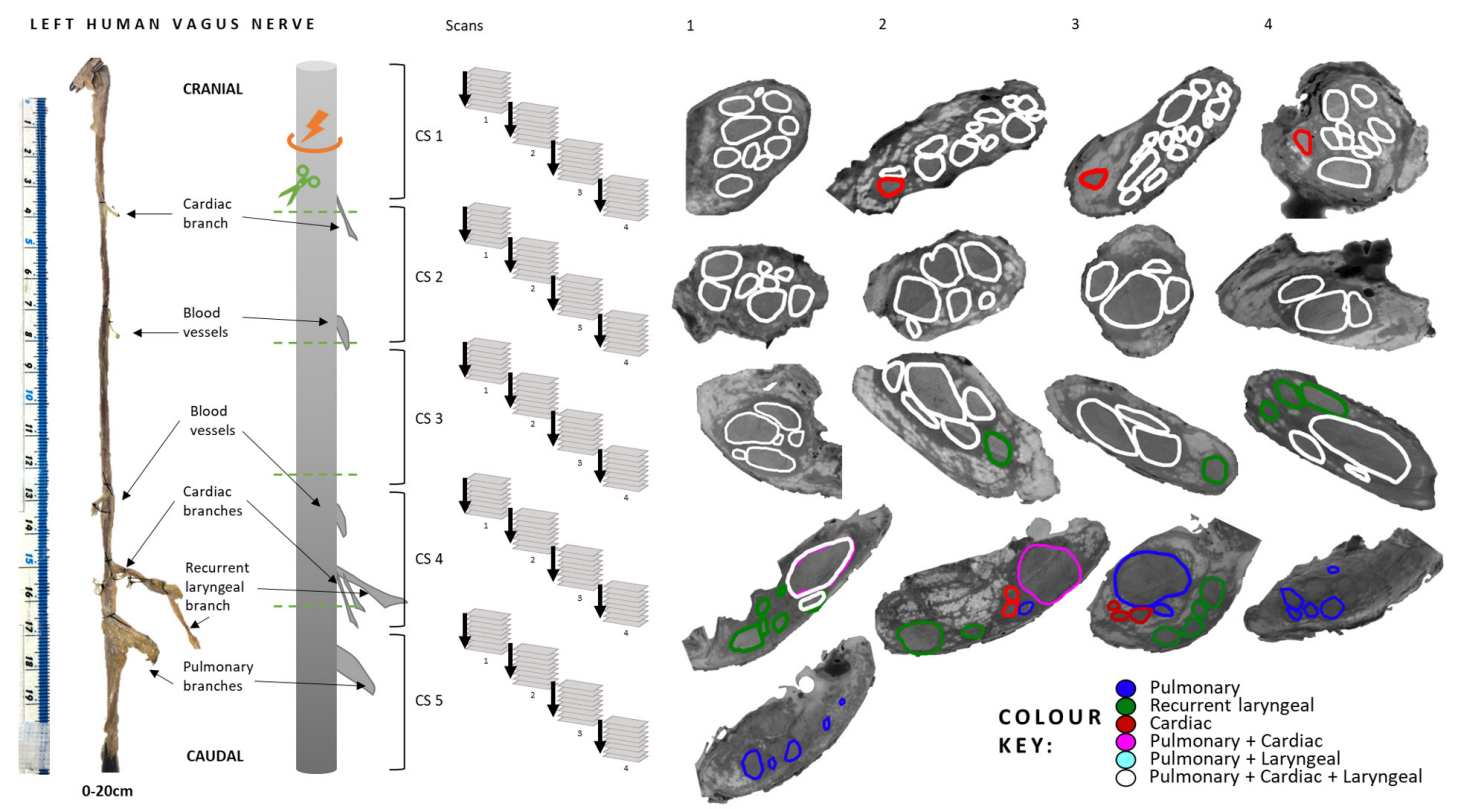
Human vagus nerve preparation, scanning setup and segmentation at intervals. The vagus nerve samples with branches marked with sutures around the trunk were cut (green dashed line) into 4 cm segments prior to staining identified here as cross-sections (CS) 1–5. The segments were bundled together and scanned with four overlapping scans. This figure illustrates how, when viewing one CS, four scans were required to image its full length. CSs from the top of each scan, proceeding cranially up the nerve from CS5 to CS1, are displayed here for one nerve example with labeled, segmented fascicles to illustrate the progression of fascicle organization and movement within the nerve.

**FIGURE 2:**
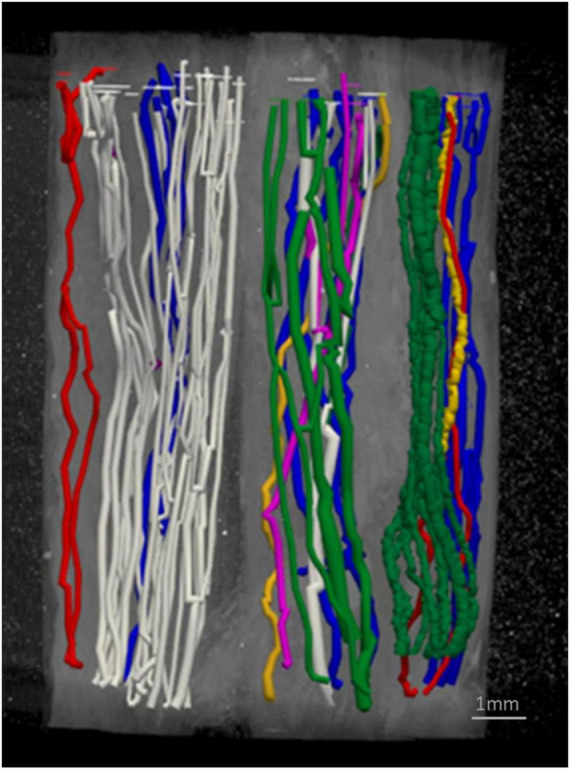
Segmentation through multiple cross-sections in a scan. An example of scan with multiple segments/cross-sections of nerve (∼1.5 cm). The fascicles have been traced through the cross-sections through the overlapping scans. Shown here is one of 4-5 overlapping scans that together encompass multiple consecutive segments of the nerve wrapped around a cylindrical sponge support. Scans were intentionally acquired with overlapping regions to enable continuous fascicle tracing and maintain traceability across adjacent scans. As a result, portions of the nerve at either end of the image appear untraced, as these regions were segmented in the preceding or subsequent scan. Colors refer to fascicles containing organ-specific fibers or a mixture of them: red—cardiac, blue -pulmonary, pink—cardiac and pulmonary, green—recurrent laryngeal, white-cardiac, pulmonary and recurrent laryngeal, yellow—cardiac and recurrent laryngeal.

**FIGURE 3:**
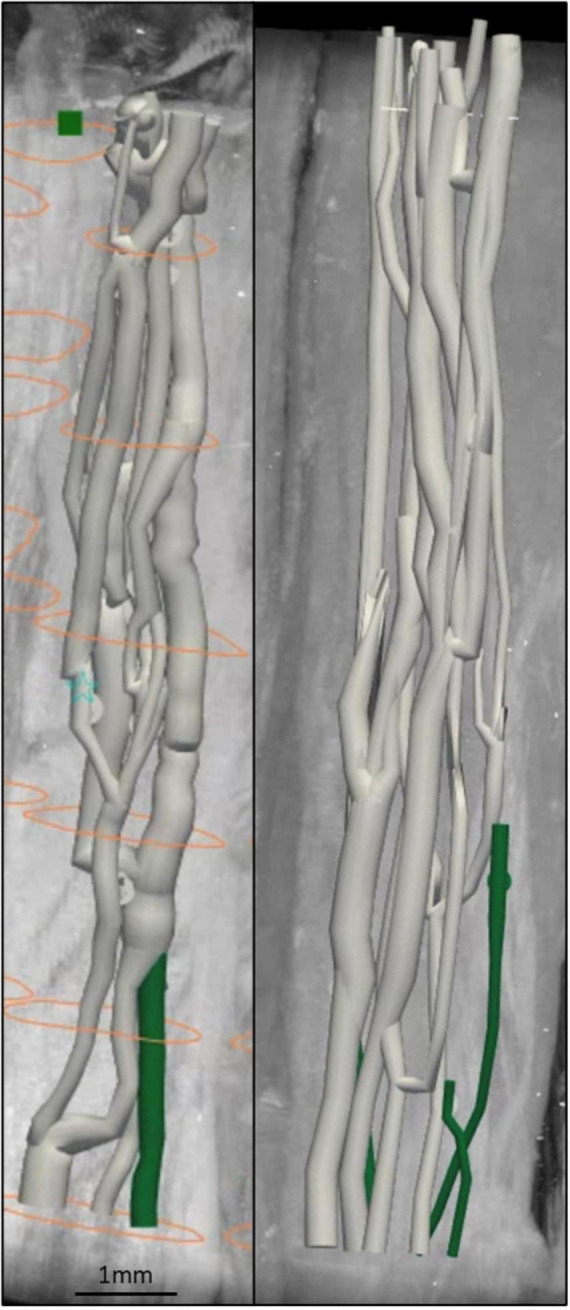
Anastomoses observed in human vagus nerve. Two examples of the frequent anastomosing (merging and splitting) between and plexiform structure of fascicles observed in the human vagus nerve in ∼1.5 cm of nerve, each showing a 2 mm diameter segment of nerve with fascicles of ranging diameter. Once organ-specific fascicles of different types merge together, the fascicular resolution of microCT scans does not allow for the tracing and identification of organ specificity after merging and during these anastomoses; therefore, from this point on, labeled as white “merged” fascicles. For another example, please see Supplementary Video 1. The orange circles in left panel depict the boundary of the nerve at intervals.

**FIGURE 4:**
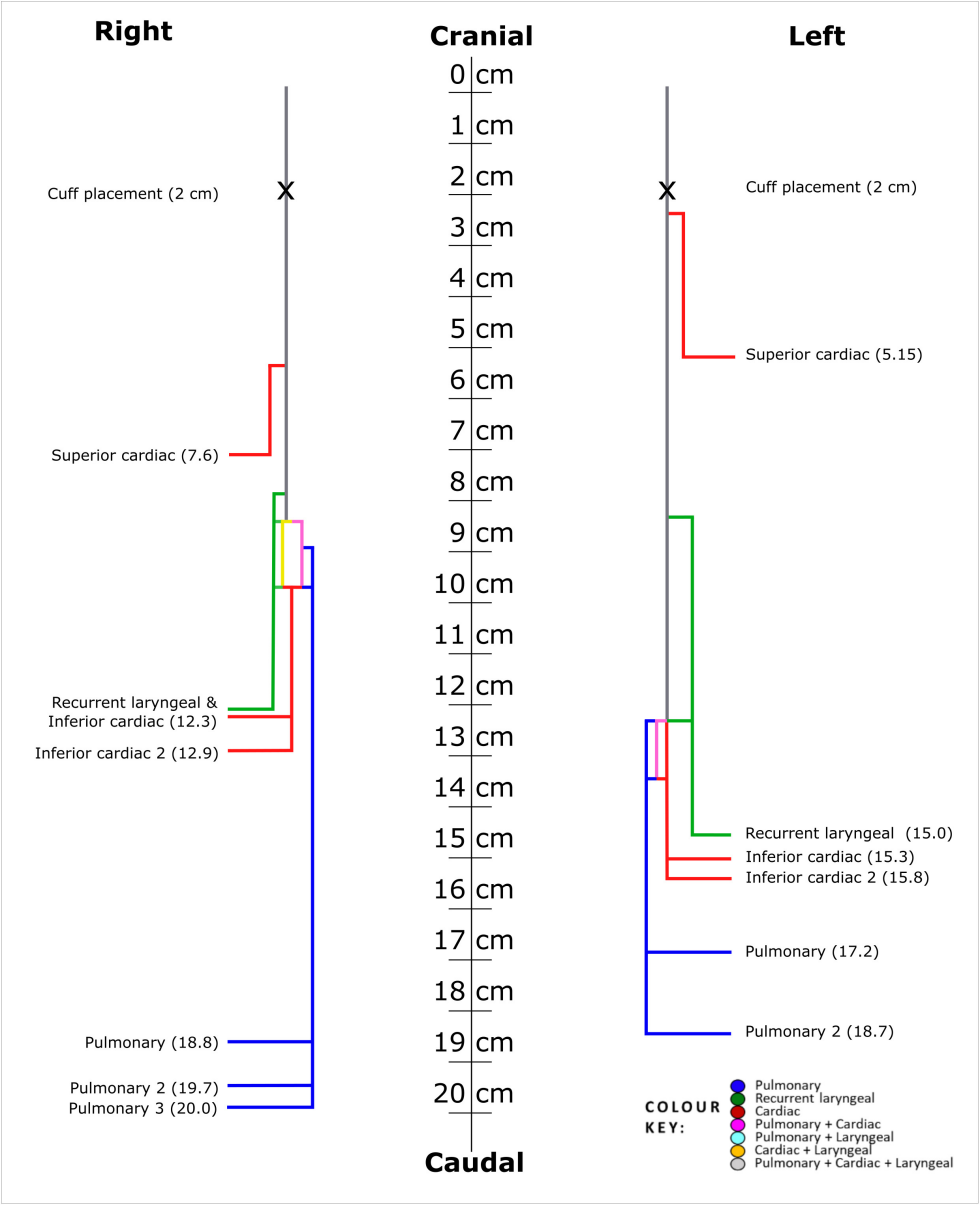
Schematic representation of fascicle organization along the cranio-caudal axis of the right and left human vagus nerves. Mean branching locations for each organ-specific branch, calculated across all nerves, are shown and used as the branch points in the schematic. Mean fascicle merge points between functional fascicle types (as reported in the Results) are also indicated. Fascicle counts and the frequency of anastomoses are not depicted. 0 cm indicates the position 2 cm cranial to the average VNS cuff placement and 5 cm caudal from the nodose ganglion.

Fascicles present in the cardiac, pulmonary and recurrent laryngeal peripheral branches were preserved after entry into the main vagus trunk (Figures 1–4 and Supplementary Figures 2–6) (*n* = 5). The organization of these organ-specific fascicles was partially preserved for approximately 1–1.5 cm. Detailed morphology of the nerve and its branches can be seen in the “Analysis of nerve morphology” section. In the left vagus nerves, approximately 1–1.5 cm cranial to branch entry, cardiac fascicles merged with some pulmonary fascicles. The remaining cardiac fascicles merged 2.5 cm cranial to branch entry point. The recurrent laryngeal fascicles remained on one half of the nerve for at least 2–3 cm and then eventually all fascicles of the recurrent laryngeal merged by 6–7 cm cranially from the branch entry point. In the right vagus nerves, the inferior cardiac not only predominantly merged with the pulmonary fascicles (forming a common, pink fascicle, Supplementary Figures 2–6) as in the left vagus nerves, but in both right vagus nerve samples segmented, the inferior cardiac fascicles merged with a recurrent laryngeal fascicle (forming a common, yellow fascicle, Figure 4 and Supplementary Figures 5, 6) at 1.7 and 2.1 cm, respectively, from point of entry in the nerve till merge point. This difference of merging pattern may be attributed to the differing branching point of recurrent laryngeal branch between left and right vagus nerves. At the point where all three organ-specific fascicle types had merged, microCT has a limitation of not allowing for the visualization and thus, the tracing of the fibers of the nerves. Therefore, the organization of the organ-specific fibers within these fascicles remains unknown. The vagus nerve structure became plexiform after all the three organ-specific fascicles merged together (Figure 3). Fascicles were observed to merge or split at an approximate rate of one to two fascicles every 0.5 mm. This measurement is based on fixed, Lugol’s-stained nerve tissue and so, shrinkage should be accounted for if translating to fresh nerve tissue. Adjusting this measurement to account for an estimated cumulative 5–15% tissue shrinkage arising from formalin fixation (approximately 5–10% for non-embedded tissue) and subsequent Lugol’s iodine staining (∼5%), the adjusted nerve length is estimated to be 0.53–0.59 mm. Further up the nerve, below the cervical level, a superior cardiac branch entered the vagus nerve and remained separated from the other fascicles for a short length (2–3 cm) which is 0.5–1 cm caudal to the VNS stimulator placement. In summary, so far, organization and separation between the organ-specific fascicular groups exists at approximately clavicular level and the superior cardiac fascicles are separate from the other vagal fascicles at a position close to VNS cuff placement.

### 3.2 Histology and immunohistochemistry

**FIGURE 5:**
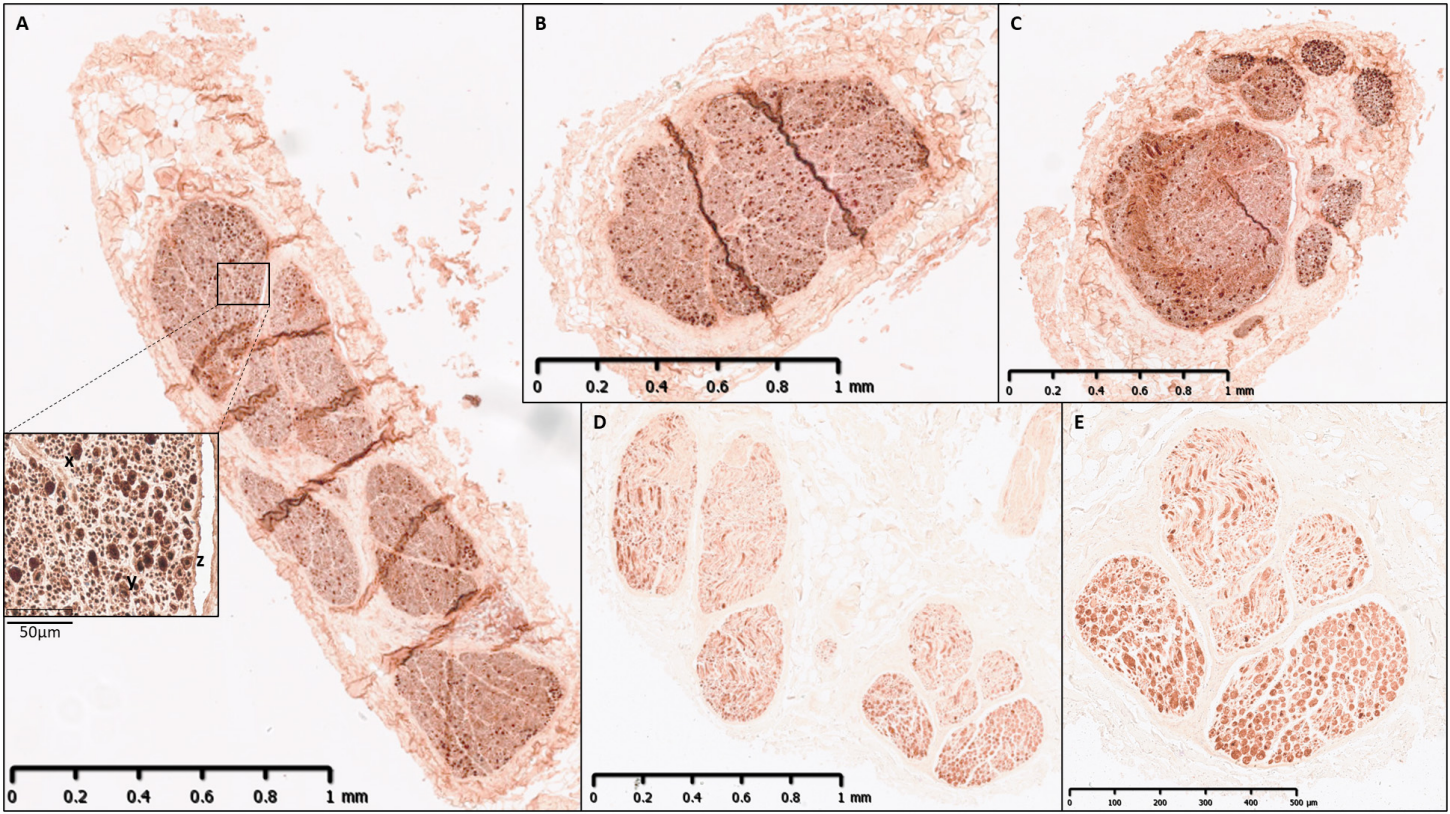
IHC of a human vagus nerve example 1. An example of strong double staining of neurofilament and myelin basic protein with cross-sections at: **(A)** Cervical level with expanded block showing distribution and dispersion of large, myelinated fibers (examples: x, y, and z); **(B)** 5 cm cranial to recurrent laryngeal branching point (one fascicle present); **(C)** recurrent laryngeal fascicles in vagus nerve trunk prior to branching; **(D)** recurrent laryngeal branch; **(E)** recurrent laryngeal fascicles (higher zoom/resolution).

**FIGURE 6:**
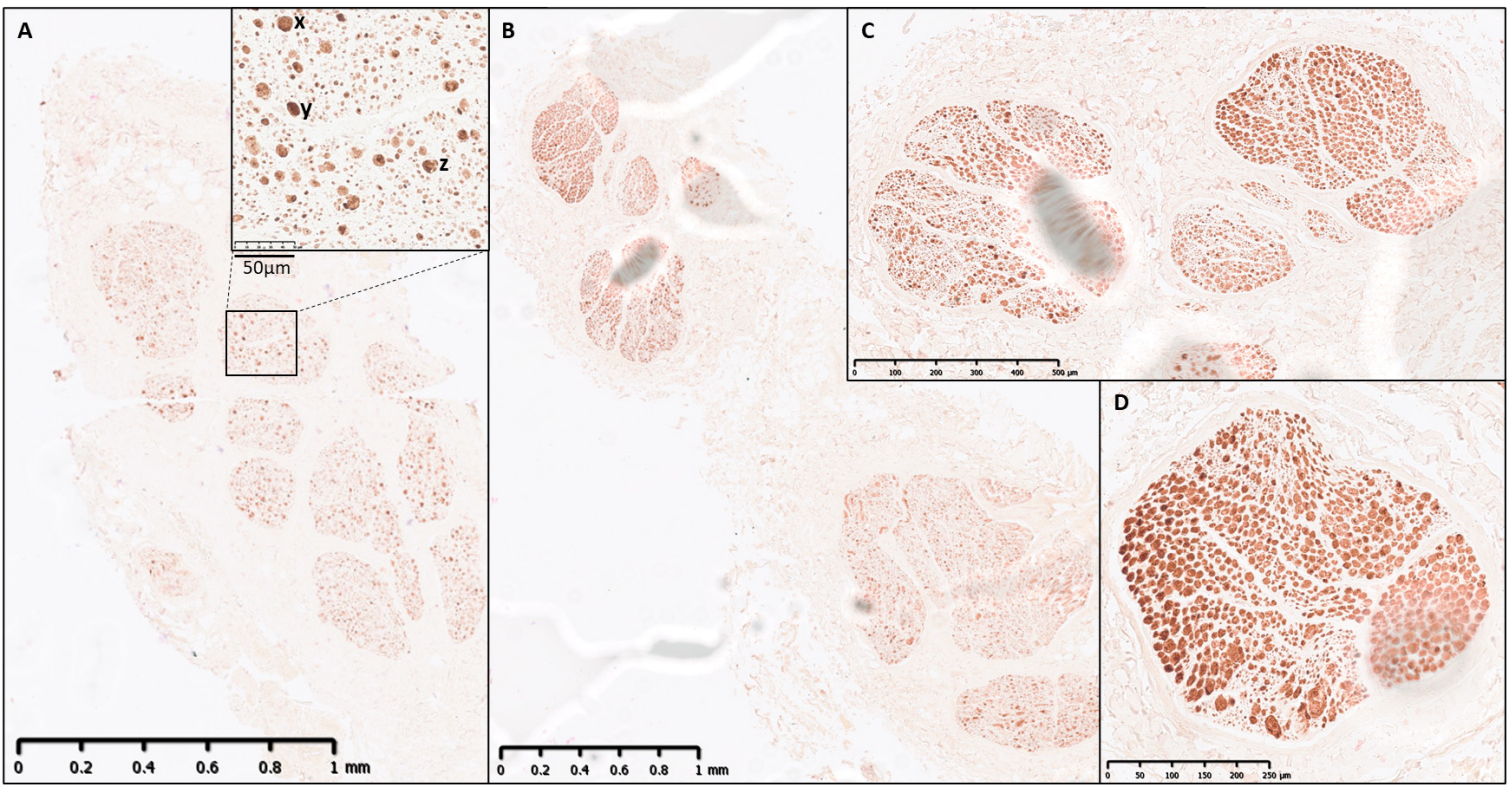
IHC of a human vagus nerve example 2. Another example of double staining of neurofilament and myelin basic protein with cross-sections at: **(A)** Cervical level with expanded block showing distribution and dispersion of large, myelinated fibers (examples: x, y, and z); **(B)** Recurrent laryngeal fascicles in vagus nerve trunk prior to branching; **(C)** Recurrent laryngeal branch; **(D)** Recurrent laryngeal fascicle (higher zoom/resolution).

Histology and immunohistochemistry were performed on all nerves including H&E, Trichrome, NF with MBP (Supplementary Figure 7). NF with MBP successfully stained fibers simultaneously, allowing clear visualization of fiber caliber heterogeneity within the cross-sections with ease (Figures 5, 6). The large, myelinated, efferent fibers in the recurrent laryngeal fascicles (Figures 5E, 6D) are evident in the cross-sections from the branch as well as in the vagal trunk just cranial to the entry of the fascicles. At the cervical level (Figures 5A, 6A), however, these large fibers appear to be mixed with smaller fibers dispersed around the cross-section and only small clusters of large fibers observed. Despite hypotheses that the clusters of fibers will remain organ-specific, this cannot be deciphered without tracing of the fibers throughout the nerve.

**FIGURE 7:**
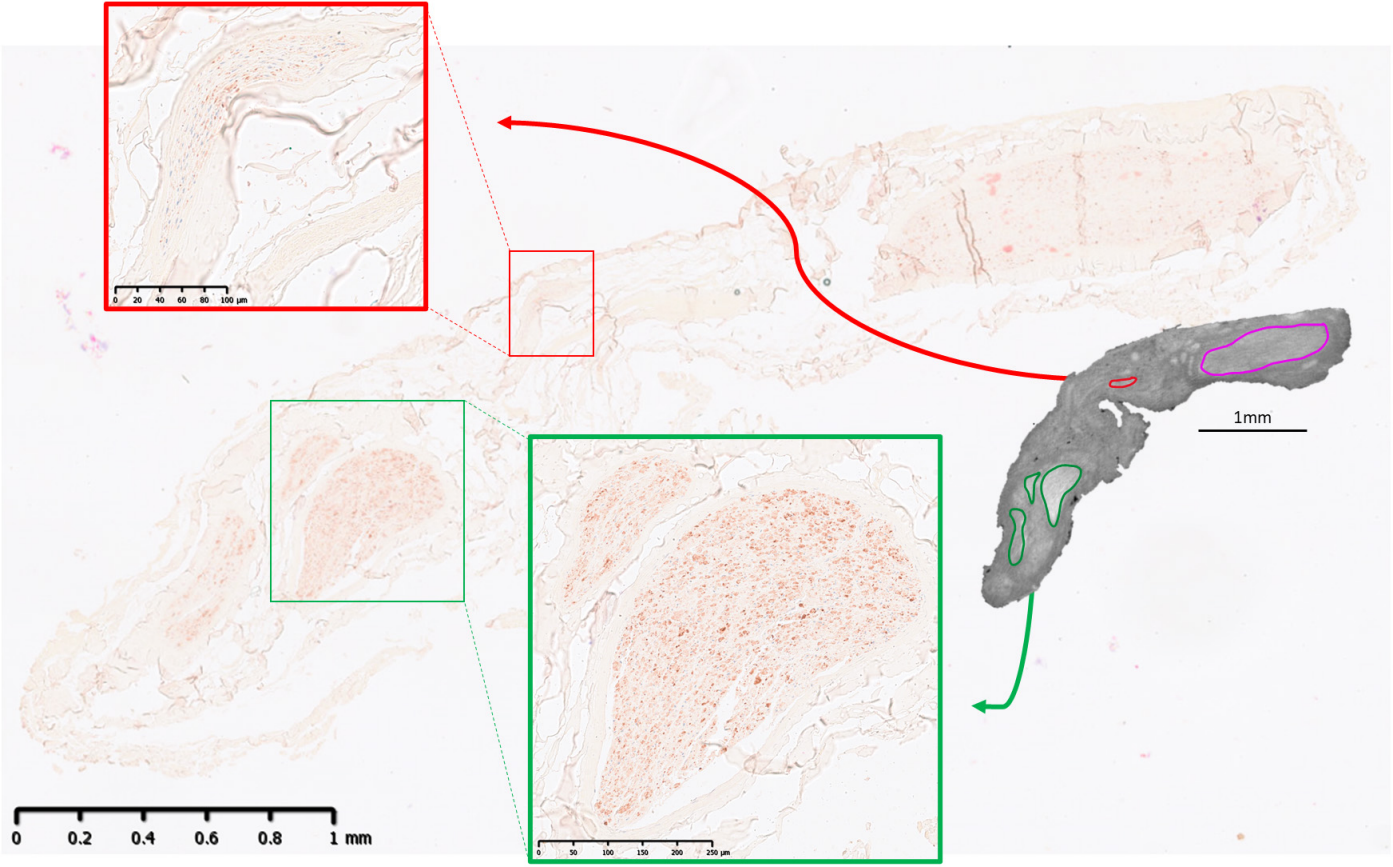
MicroCT with IHC 1. An example from one nerve of IHC compared to microCT 14 cm from mid-cervical level with cardiac (red) and recurrent laryngeal (green) fascicles highlighted. A large merged fascicle between cardiac and pulmonary (pink) are also visible in the microCT cross-section.

**FIGURE 8:**
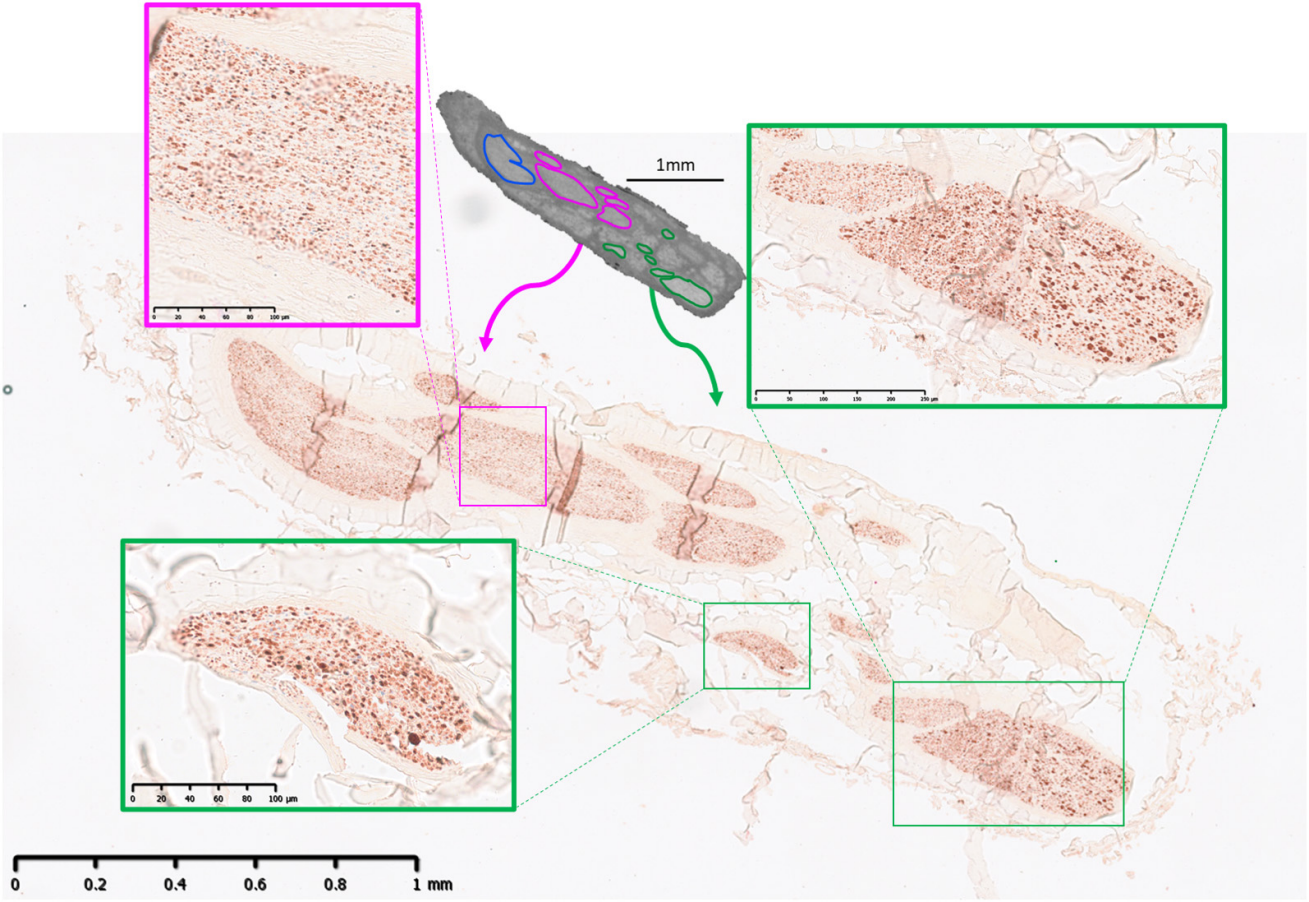
MicroCT with IHC 2. An example from one nerve of IHC compared to microCT 13 cm from mid-cervical level with merged between cardiac and pulmonary (pink) and recurrent laryngeal (green) fascicles highlighted. Pulmonary (blue) fascicles are also visible in the microCT cross-section.

**FIGURE 9:**
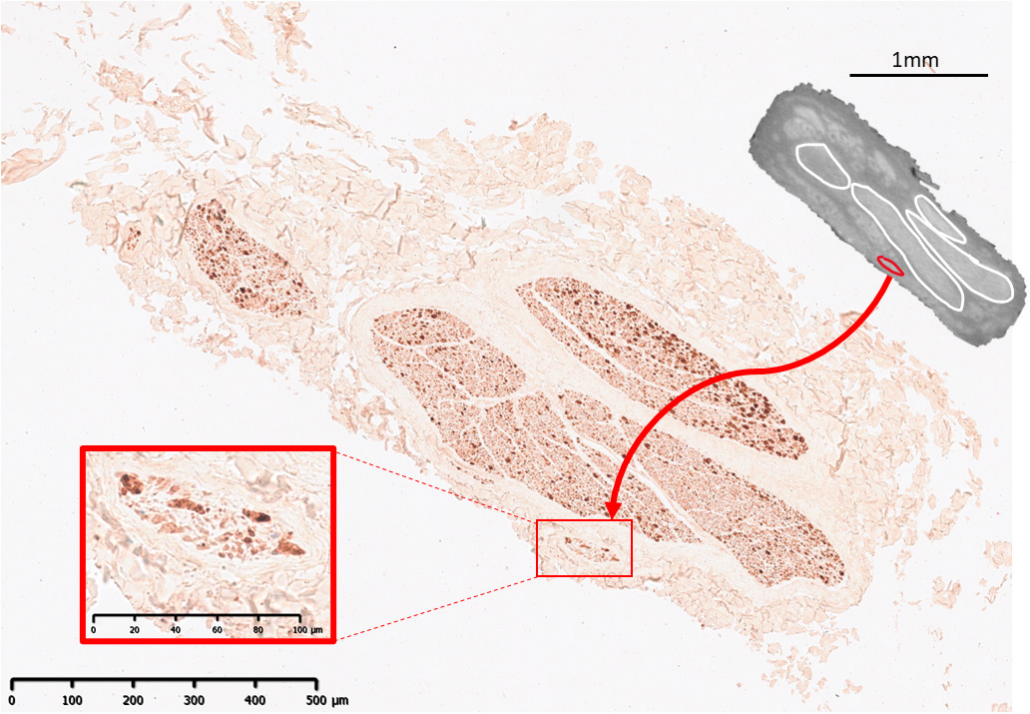
MicroCT with IHC 3. An example from one nerve of IHC compared to microCT 7 cm from mid-cervical level with a superior cardiac fascicle (red) highlighted. Merged fascicles between inferior cardiac, recurrent laryngeal and pulmonary (white) are also visible in the microCT cross-section.

Histology and immunohistochemistry allowed for validation of microCT segmentation—confirming the presence of fascicles from that which was segmented from the scans. A nerve example of microCT compared with immunohistochemistry can be seen in Figures 7–9. Regions of the stack of XY slices of microCT data from where the histology slice was taken were compared to the histology slice of interest to find the matching slice for accurate cross-validation. For display purposes here, the closest already segmented and labeled cross-section was compared and therefore, there is slight mismatch. Recurrent laryngeal fascicles can be seen to contain more larger, myelinated fibers than the other fascicles in the nerve. The mixed (cardiac and pulmonary) fascicles contained a mix of fiber sizes. The superior cardiac (Figure 9) fascicle also contained a mixture of fiber sizes.

### 3.3 Analysis of nerve morphology and anatomy from microCT and histology

From the morphological data of the nerve (*n* = 10), the right human vagus nerves were 1.22 ± 0.1 SD times larger than the left vagus nerve in terms of its diameters, circumference, and area, with an average area of all 10 nerves of 3.63 mm^2^ ± 1.43 SD (variance (var) = 1.84, 95% CI [2.61, 4.65]), an average area of the left vagus nerves of 3.08 mm^2^ ± 1.77 SD [var = 2.51, 95% CI (0.88, 5.28)], and an average area of the right vagus nerves of 4.17 mm^2^ ± 0.85 SD [var = 0.57, 95% CI (3.12, 5.22)]. In addition, the right vagus nerves contain 1.35 times more fascicles and fascicle bundles than the left: 10.80 ± 4.64 SD [var = 19.36, 95% CI (7.48, 14.12)], 9.60 ± 3.05 SD [var = 7.44, 95% CI (5.81, 13.39)], and 12.00 ± 5.96 SD [var = 28.40, 95% CI (5.00, 19.8)] for all, left and right fascicles, respectively (Supplementary Tables 3, 4 and Supplementary Figure 8). The left and right cervical vagus nerves consisted of 5.00 ± 1.58 SD [var = 2.00, 95% CI (3.04, 6.96)] and 7.20 ± 3.70 SD [var = 10.96, 95% CI (2.6, 11.8)] fascicle bundles, respectively.

The number of fascicles present in the cross-sections of the main trunk of the vagus nerve varied when progressing through the nerve at regular intervals (Supplementary Tables 3, 4). The least number of fascicles was 8.30 ± 3.43 and 8.67 ± 4.5 at 1 cm and 13 cm from the upper cervical level, respectively. At 9 cm from upper cervical level, there was the greatest average number of fascicles across the 10 nerves of 16.20 ± 7.28. Such changes in fascicle counts when progressing down the length of the nerve illustrate the anastomoses taking place. This coincides with the high standard deviations observed here such as a standard deviation of 10.19 for nerve 3. The mean fascicle count along the entire nerve length was 11.85 ± 5.75. This is greater than the mean at cervical level. On average between nerves, the superior cardiac branch contained 3.10 ± 1.45 fascicles, the inferior cardiac branch 2.33 ± 1.06, recurrent laryngeal branch 7.25 ± 1.98, and the pulmonary branch 6.14 ± 2.91 fascicles.

Unlike the pig vagus nerves, the human inferior cardiac branches exit the vagus nerve just above or at the same level as the recurrent laryngeal branches (Supplementary Table 5). The distance from cervical level on average to the inferior cardiac branches and recurrent laryngeal branch was 12.30 cm ± 1.89 and 12.30 cm ± 1.89, and 15.30 cm ± 1.44 and 15.00 cm ± 1.27 for the right and left nerves, respectively. The discrepancy of the branching point of the right and left recurrent laryngeal branches of the vagus nerve is expected. The superior cardiac branch exited the vagus nerve 6.38 cm ± 2.43 from the upper cervical level with a difference of 2.45 cm between the left and right nerves with the branch exiting at 5.15 cm and 7.60 cm, respectively. The pulmonary branches exited the vagus nerve from 18 to 20 cm from the upper cervical level. Four fifths of the right vagus nerves had three pulmonary branches and four fifths of the left vagus nerve has two pulmonary branches which coincides with the number of lobes the lungs consist of on either side. There was a difference of 1.2 cm ± 0.71 and 0.70 cm ± 0.27 between the first and second, and between the second and third pulmonary branches, respectively.

Compared to morphological data previously reported in literature, the effective diameters were comparable between studies (Table 1). In addition, the fascicle counts, when looking at fascicle bundles in this work, was comparable between studies too. The individual fascicle count reported here was larger; this may be indicative of the other studies not taking into account thin layers of perineurium. The nerve cross-sectional areas correlated with Pelot et al. (2020) but the other three studies reported greater areas of 1.5 times, albeit with overlapping ranges.

## 4 Discussion

In summary, the segmented human vagus nerves showed a fascicular, organotopic organization from entry of the peripheral cardiac, pulmonary and laryngeal branches up until clavicular level. Thereafter, the fascicles anastomosed and formed a plexiform structure within the nerve from clavicular level until cervical level with a frequency of one to two fascicles merging or splitting every 0.5 mm. The superior cardiac fascicle(s) are still separate from the rest of the merged vagal fascicles at level of cuff placement (*n* = 3, 1 left, 2 right) or 0.5–1 cm caudal to cuff placement (*n* = 2 left) still within the mid-cervical level in a readily accessible location for cuff placement. The merging pattern between fascicles of different organ origin was reasonably similar between nerve samples and between sides. However, there was one notable difference between left and right: the inferior cardiac branch fascicles of the right vagus nerve not only merged predominantly with the pulmonary fascicles, as on the left side, but one fascicle also merged with a recurrent laryngeal fascicle. This difference of merging pattern between sides may be attributed to the different average location of the recurrent laryngeal branching point on the vagus. On average, at cervical level, the left vagus nerve contained 9.6 and 5 and the right contained 11.6 and 6.8 fascicles and fascicle bundles, respectively. The cross-sectional areas of the right and left vagus nerves were 4.17 mm^2^ ± 0.85 and 3.08 mm^2^ ± 1.77, respectively. The number of fascicles comprising the vagus nerve at regular intervals varied significantly, increasing and decreasing along its length with only an observed pattern of the most fascicles being present at 9 cm caudal from mid-cervical level on average across ten nerves. Histology allowed for validation of the microCT segmentation and allowed for visualization of fiber size dispersion and clusters present in the types of fascicles in the nerve. The large fibers observed in recurrent laryngeal fascicles from its branch appear to be dispersed around fascicles of the nerve with only small clusters still remaining at cervical level.

The morphometric data was comparable with previously reported data in terms of diameter, area and fascicle counts (Hammer et al., 2018; Pelot et al., 2020; Stakenborg et al., 2020; Verlinden et al., 2016; Table 1). The cross-sectional areas reported in three of the studies was greater than those reported here and in Pelot et al. (2020). However, the diameters were similar. This may indicate differences in calculations of area between studies. These data together should be used as an informative dataset for human cervical vagus nerve morphology which could inform cuff design, stimulation strategies and cuff placement. Averaging the five studies’ data, the human cervical vagus nerve has an area of 5.49 mm^2^ ± 2.07, a diameter of 2.47 mm ± 0.56, and 6.25 ± 1.89 fascicles.

The frequency of splitting and merging (anastomosing) correlated with that previously quantified over 2 cm of cervical vagus nerve (Upadhye et al., 2022). This also corresponds with the large variance in fascicle counts along the length of the nerve at regular intervals observed for all ten nerves. The high degree of fascicular anastomoses along with the variations in key morphological characteristics of fascicles across humans may attribute for the dispersion of fibers observed at cervical level compared to within fascicles of branches, and even further, could potentially account for the diverse clinical responses seen in patients undergoing VNS.

**FIGURE 10:**
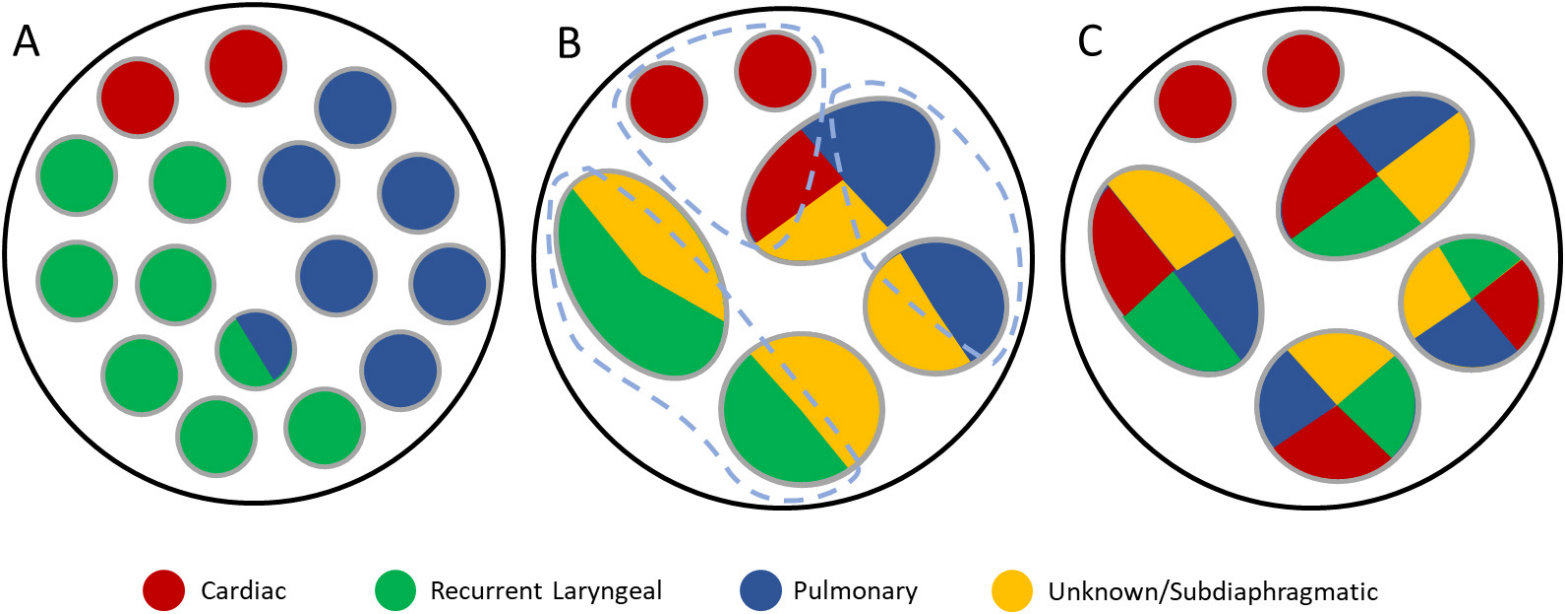
Didactic illustration of organization in the cervical vagus nerve of pigs and humans. **(A)** Observed pig vagus nerve organization of cardiac, pulmonary and recurrent laryngeal fiber-containing fascicles (Thompson et al., 2023). **(B)** A potential organization of human cervical vagus nerve fascicles where there is spatial organization of fibers within the fascicles across the overall cross-section of the nerve, still allowing for selective activation of organ-specific fiber groups (encircled in gray dashed lines) despite distribution across multiple fascicles. **(C)** A second potential organization of human cervical vagus nerve fascicles where there is no spatially separated localization of fibers across the total cross-section which would prove difficult to perform spatially-selective stimulation of a specific group of organ-specific fibers.

The dispersion of large fibers across the cross-section and fascicles at the mid-cervical level, seen in the IHC cross-sections, is unexpected. No tracing of fibers was performed, and there are still clusters of larger fibers visible at the cervical level. There may still be organization of organ-specific fibers at this level. This would be expected considering the pig vagus nerve was organized organotopically with respect to the three organs (Figure 10A), and human somatic nerves are organized somatotopically (Bäumer et al., 2015; Stewart, 2003; Zill et al., 1980). On the other hand, the nodose ganglia house the cell bodies of the afferent fibers and the DVMN the efferent cell bodies. If the vagus nerve was reorganizing itself from organ-specific to fiber-specific, one would expect the clusters of larger fibers to be more pronounced at the cervical level and not dispersed. Tracing of fibers needs to be performed to provide these answers.

The fascicular anatomy of vagus nerve is highly complex and dynamic, and it appears even more so in the human vagus nerve. Mapping nerves using microCT is an effective technique to visualize and quantify fascicular organization. Understanding the nerve morphology and anatomy can assist in improving electrode cuff design as well as stimulation strategies. Given that humans have three to five times fewer but larger fascicles compared to the pig, their fibers are expected to have higher activation thresholds (Grinberg et al., 2008; Pelot et al., 2017). These analyses of fascicle dynamics, anatomy and organization within the human cervical vagus nerve provides further perspective into the morphology of the nerves and its implications on VNS efficacy. This data can contribute to computational modeling and *in vivo* studies for investigation of improved selectivity and vagal neuromodulation techniques. This will ultimately allow for improvement of the efficiency of treatment and the patients’ quality of life.

A clear limitation of this study was the fascicular resolution of the microCT scans which only allows for accurate and confident tracing of organ-specific fascicles prior to merger of other known organ-specific fascicles. This limited the tracing of organ-specific fascicles beyond clavicular level; however, microCT with segmentation still provided valuable information.

Specifically, microCT with iodine staining identifies fascicles from the rest of the soft tissue of the nerve; it does not enable visualization of individual nerve fibers within the fascicles. In this work, we employed the following principle for assignment of function to cranial cervical fascicles. We identified the function of a distal peripheral branch by its proximity to the supplied end organ. We then traced the branch and respective fascicles cranially. Providing the fascicles either did not diverge cranially, only converged cranially, or merged with the same fascicle type and subsequently diverged cranially, it was logical to infer that the assigned organ function was unique. However, if two or more fascicles supplied by different organs merged and then diverged cranially, it was unclear which organ function was subserved in the cranial diverged fascicles. It is possible that each fascicle was still organ specific, but it may also have been the case that nerve fibers had mixed so that each divergent cranial fascicle now was supplied by more than one organ. In either case, this organ supply cannot be identified from fascicle tracing alone. Based on the hypothesis that fibers pass between fascicles during this cross-over, when fascicles of two organ-specific types merged, the resulting merged fascicle was labeled as a mixed according to the organ-specific fascicles of origin. If that fascicle then continued cranially and diverged again, the resulting fascicles would maintain that mixed fascicle label. This erred on the side of caution as it may have been that some cranial divergent fascicles were actually still organ-specific. If all fascicles merge proceeding cranially, all resulting fascicles will be labeled as mixed fascicles.

Despite this limitation, by performing this microCT and tracing method, the following could be determined, quantified, analyzed, and subsequently compared between individuals: (1) the path of the fascicles within the nerve from entry into the vagus till cervical level, (2) the arrangement of fascicles at the cervical level, (3) the general pattern of fascicle movement, anastomoses and pathway in the nerve, (4) the number of fascicles for each branch and within the length of the vagus, (5) the number of and distances between branches to specific organs and effectors (general gross vagal anatomy), (6) the distances between first and last merging of fascicles of a specific organ-type within the vagus i.e., distances fascicles travel in a cable-like structure before merging with other fascicle types (fascicular morphology), and (7) the frequency of merging and splitting between fascicles of the same type and of different types i.e., the frequency of cross-over between fascicles, the plexiform structure of the nerves.

Other methods could be used to provide either fiber-specific tracing of the vagus nerve such as iDISCO (Renier et al., 2014), 3D-MUSE (Kolluru et al., 2021), or painstaking fiber tracing from serial IHC, or functional imaging of areas in the nerve with EIT or selective stimulation. Another limitation was not including the nodose ganglion in the dissections of the human vagus nerve. This would have allowed for tracing of fascicles bypassing the nodose, which would contain efferent fibers, and those originating from the nodose, which would contain afferent fibers. It may also have resulted in merger of fascicles and the same issue as above; however, this was indeed a short sight.

Performing histology and IHC at regular intervals allowed for morphological analyses and comparison with other studies. Additionally, this work served as protocol development for future human IHC studies and fiber analyses. Even by performing this at intervals as shown here, this will allow for calculation of the total number and ratio of fibers in the nerve, and in specific fascicles, and how this changes/shifts in proportion progressively at regular intervals in the nerve, with respect to efferent/afferent, myelinated/unmyelinated, and parasympathetic/sympathetic. In addition, it will be possible to determine the diameters of the fibers, and thereby, the numbers of various fiber types (A, B, C, etc.). If these methods were to be performed on sequential slices throughout the nerve the following could be determined: the path of the vagal fibers of the organ-specific fascicles from entry point in the vagus nerve to beyond the nodose ganglion to the brainstem. Specifically, the fibers could be traced throughout the merger and subsequently split of fascicles when progressing up the nerve (i.e., tracing organ-specific fibers, not fascicles, even when and after fascicles of various types merge and microCT cannot discern fascicle types, besides tracing a “merged” fascicle for the rest of the nerve). In addition, a fiber map for all fascicles could be delineated at the cervical level and throughout the vagus nerve length, specific to organ and fiber type (efferent/afferent, presence of any hitchhiking sympathetic, etc.). Lastly, fiber tracing and microCT fascicle tracing a could be compared to determine any patterns that can be deduced/inferred from one to the other.

Choline Acetyltransferase (ChAT) is the enzyme that is responsible for biosynthesis of the neurotransmitter acetylcholine. The majority of acetylcholine is synthesized locally at nerve terminals where ChAT catalyzes the transfer of an acetyl group from acetyl coenzyme A to choline, a process that takes place in a single step. ChAT is expressed by cholinergic neurons in the central and peripheral nervous systems (Oda, 1999). It is a general parasympathetic cholinergic-specific histochemical stain but has different sensitivities to motor (efferent) vs. sensory (afferent) fibers; a study showed it is present predominantly in motor and scantly in sensory in a ratio of 8:1 (Agarwal et al., 2020). ChAT has been used frequently to differentiate between efferent and afferent fibers (Agarwal et al., 2020; Settell et al., 2020; Zhou et al., 2021). With immunohistochemical markers such as this, and sometimes in a combination, the various neuron types can be identified. Specifically, motor markers include ChAT, cholinesterase, tyrosine kinase (TrkA), and agrin whilst sensory markers include calcitonin gene-related peptide (CGRP) and transient receptor potential vanillic acid subtype 1 (TRPV1), and annexin V (Agarwal et al., 2020; Meng et al., 2011; Riley et al., 1988; Zhou et al., 2021). Sympathetic fibers can be distinguished from parasympathetic by using tyrosine hydroxylase (Weihe et al., 2006). While many studies have used various combinations of IHC markers to distinguish fibers, such as motor vs. sensory, no method seems to be standardized or largely adopted with new method development studies as recent as this year (2026). However, it seems probable to develop a combination of IHC stains to provide informative anatomical characterization of parasympathetic and, specifically, vagus nerves.

Future work could include fiber analysis of the cross-sections at regular intervals and from the branches including quantifying and measuring the fiber sizes, types, ratios, and their organization across the slice, and lastly, how this changes/shifts in proportion progressively at regular intervals in the nerve. It needs to be determined whether the fascicles at cervical level are still somewhat organized in a manner consistent with the pig vagus nerves (Figure 10A) and the human somatic nerves with fibers localized into a region of the whole vagus nerve cross-section, such as in Figure 10B, which would still allow for spatially selective stimulation of organ-specific fibers, or if there is little spatial organization of organ-specific fibers across the cross-section at cervical level at all as in Figure 10C. ChAT staining should be optimized for human nerves in the future. This would provide insight into the distribution of efferent fibers within the fascicles of the nerve. Additionally, double staining with NF and MBP should be done with fluorescence. This will allow for easier and more efficient visualization of the neuron versus the myelin sheath and will allow for quantification of numbers and sizes with readily available software that can detect fluorescence with ease. Tyrosine hydroxylase could also be detected for to see if any sympathetic fibers are present in the vagus nerve. Additionally, in order to determine functional organization in human vagus nerves, EIT and selective stimulation is being used in an ongoing *in vivo* clinical study. This will allow for localization of organ-specific functional activity within the cervical vagus nerve, and thereby determination of possible organization beyond structural imaging methods, and could ultimately allow for a patient-specific map to guide targeted stimulation for each individual.

## 5 Conclusion

Similar to the somatic nervous system and the organotopic organization observed in pigs, it seems reasonable to hypothesize that despite organ-specific fascicles merging and anastomosing throughout proportions of the human vagus nerve, fibers within merged fascicles in the human vagus nerve at cervical level could still be somewhat arranged according to their supply to individual organs and possibly specific functions. Studies *in vivo* in human patients testing the electrophysiology of the nerve, stimulating spatially separated regions of the vagus and observing physiological readouts for different organ function, such as EIT and selective stimulation in Thompson et al. (2023) could assist with providing information beyond the limits of the fascicular resolution of microCT.

The superior cardiac fascicles remained separate at level of cuff placement which shows promise for selectivity of neuromodulation of the heart. This could benefit a number of conditions for which pharmaceuticals and other treatments are not sufficient in some cases, such as myocardial infarction, heart failure, atrial fibrillation (De Ferrari and Schwartz, 2011; Sabbah et al., 2011; Yao and Kothare, 2020).

Here, we have provided novel morphological information of human vagus nerves to contribute to the minimal knowledge currently available and so contributing to the currently insufficient anatomical dataset that could assist with improvement of electrode cuff designs, stimulation strategies, and improvements to selective VNS and thereby accelerate the development of novel neuromodulation therapies for autonomic regulation. Furthermore, this research has implications for improving the clinical applications of VNS, nerve repair, and regeneration, ultimately leading to more precise and effective therapeutic interventions. Additionally, the preliminary investigation of histological and IHC analyses contribute to further understanding of anatomy and morphology of the vagus nerve and serve as a foundation and step toward future advanced fiber tracing studies.

## Data Availability

The original contributions presented in this study are included in this article/Supplementary material, further inquiries can be directed to the corresponding author.
