# Supplementary material for: Human vagus nerve fascicular anatomy and its implications for targeted cardiac stimulation: a microCT segmentation and histological pilot anatomical study
Source: Front Neurosci. 2026 Feb 18;20:1731234. doi: 10.3389/fnins.2026.1731234 (PMC12956647; doi:10.3389/fnins.2026.1731234)
Supplement: Supplementary file 1 [file Data_Sheet_1.pdf]

SUPPLEMENTARY INFORMATION

Human vagus nerve fascicular anatomy and its implications for targeted cardiac stimulation: a microCT segmentation and histological pilot anatomical study

Nicole Thompson[1]*, Svetlana Mastitskaya[1], Francesco Iacoviello[2], Felicia Turhani[1], Paul R. Shearing[2], Kirill Aristovich[1], David Holder[1]

[1] Department of Medical Physics and Biomedical Engineering, University College London; Gower Street, London WC1E 6BT, United Kingdom

[2] Electrochemical Innovations Lab, Department of Chemical Engineering, University College London; Gower Street, London WC1E 6BT, United Kingdom

*** Correspondence:**
Nicole Thompson
nicole.thompson@ucl.ac.uk

Supplementary Table 1. Cadaver and nerve information

Cadaver	*Nerves*	*Age*	*Sex*	*Cause of death*	*Cadaver storage temperature*	*Fixation duration pre Lugol's staining*	*Fixation duration post Lugol's staining*	*Nerve storage temperature*
A	1, 2	88	F	Myocardial Infarction	-20° to -15°C	>7 days	>7 days	4°C
B	3, 4	68	F	Septic Schock	-20° to -15°C	>7 days	>7 days	4°C
C	5, 6	77	F	Lung Mass, Presumed Lung Cancer	-20° to -15°C	>7 days	>7 days	4°C
D	7, 8	56	F	Adenocarcinoma of Duodenum	-20° to -15°C	>7 days	>7 days	4°C
E	9, 10	60	F	Aneurysm	-20° to -15°C	>7 days	>7 days	4°C

IHC Protocol

1. Immerse in TBS buffer (Sigma 94158) or BOND Wash Solution (Leica Biosystems AR9590) – here on out referred to as TBS buffer
2. Immerse in hydrogen peroxide in TBS for 15 minutes
3. Wash slides with TBS with 3 changes of 2 minutes each
4. Immerse in 5% donkey serum (Sigma D9663) for 30 minutes
5. Rinse with water
6. Add diluted primary antibody(ies) for 1 hour (NF and MBP together)
    a. For NF: anti-neurofilament heavy polypeptide antibody ab8135 1:1000
    b. For MBP: anti-myelin basic protein antibody ab7349 1:4000
7. Wash slides with TBS with 3 changes of 2 minutes each
8. Add secondary biotin labelled antibody diluted in TBS 1:1000 for 1 hour (donkey anti-rabbit IgG H&L (Biotin) ab6801)
9. Wash slides with TBS with 3 changes of 2 minutes each
10. Add tertiary streptavidin/biotin complex in TBS for 30 min
11. Wash slides with TBS with 3 changes of 2 minutes each
12. Add DAB solution (Sigma D3939) for 5 minutes
13. Wash with distilled water
14. Add secondary HRP labelled antibody diluted in TBS 1:1000 for 1 hour (donkey anti-rat IgG H&L (HRP) ab102182)
15. Wash slides with TBS with 3 changes of 10 minutes each
16. Add Vector NovaRed Substrate Kit (Vector Laboratories SK-4800) tertiary solution in TBS for 30 min
17. Wash slides with TBS with 3 changes of 10 minutes each
18. Add the Vector NovaRed substrate for 5 minutes
19. Wash with distilled water
20. Add Harris's hematoxylin (diluted 1:1) for 1 minute
21. Wash with water for 3 minutes
22. Dehydrate through alcohols into xylene
23. Remove from the IHC machine and place in water
24. Coverslip slides with DPX

Matching neighboring nerve segments

The nerve was cut into 4 cm segments subsequent to the placement of sutures as landmarks across the cut region. The number of suture landmarks differs between the cut regions to ensure the correct neighboring segments were matched up (an additional measure to the nerve segments being arranged in the correct order). The last whole cross section visible in the last scan of a segment of nerve was identified and visualized with the first whole cross section visible in the first scan of the next segment of nerve. The suture landmarks were identified and the cross sections' orientation aligned to allow for further correlation of fascicles across the cut region. The size, number, position and pattern of fascicles were matched up between the two cross-sections of each cut region between nerve segments (Supplementary Figure 1). Additional anatomical landmarks, such as blood vessels, connective tissue or fat cells were also used to assist in ensuring correct correlation between cross sections. Occasionally, fascicles were in the process of merging or splitting across the cut region (three encircled examples are shown in the cross sections of Supplementary Figure 1); however, with process of elimination with the other identified and matched fascicles, and viewing the scans till the respective ends where the whole nerve may not be visible, but the slight movement of fascicles of interest is, it can be deduced that these fascicles follow on from one another. The identified merged fascicle or split fascicles will be incorporated in the continuation of segmentation.

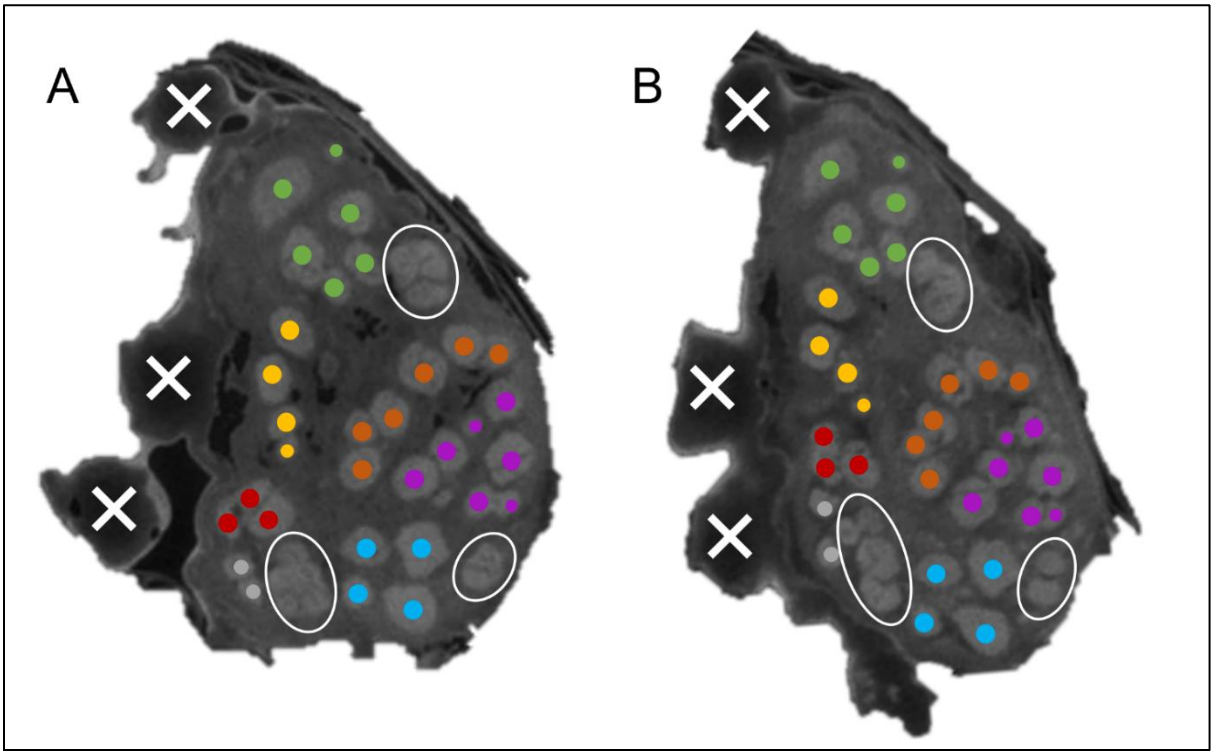

Supplementary Figure 1. Cross sections from neighboring cut segments of pig nerve (Thompson et al., 2023). An example of co-registration between cut regions of pig nerve – the same process was used in this study with human nerve. The last whole cross section visible in

the last scan of a segment of nerve (**A**) is matched up to the first whole cross section visible in the first scan of the next segment of nerve (**B**). Suture landmarks were used to orient the nerve initially – shown here with white crosses. The number of suture landmarks differs between the cut regions to ensure the correct neighboring segments were matched up (an additional measure to the nerve segments being arranged in the correct order). The size, number, position and pattern of fascicles were matched up between the two cross-sections – shown here in various color groupings for ease of visualization. The three areas encircled in white here depict fascicles that were in the process of merging or splitting across this cut region; however, with process of elimination (with the other fascicles identified as matching) and viewing the scans till the respective ends, it can be deduced that these fascicles follow on from one another.

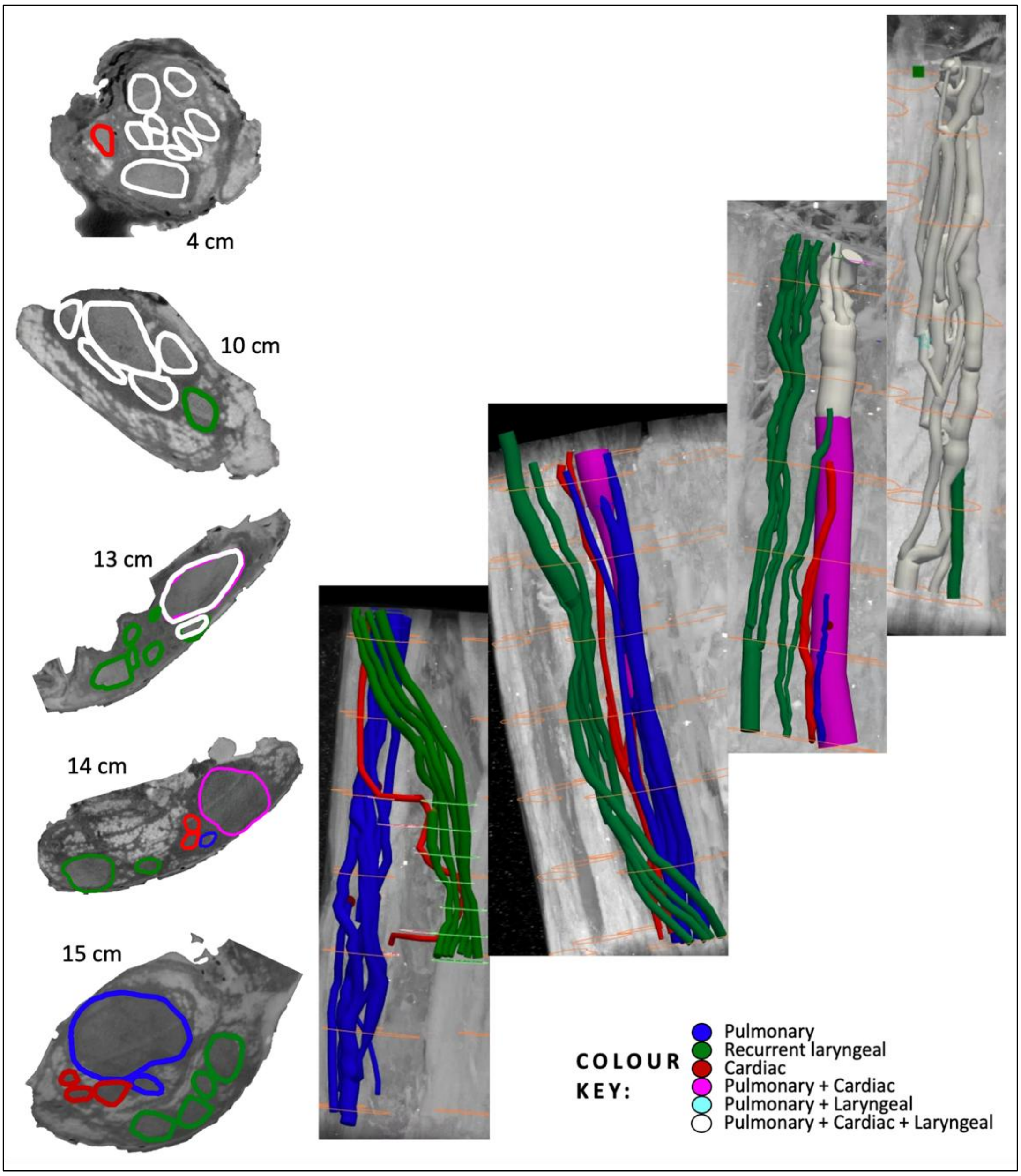

Supplementary Figure 2. Human vagus nerve segmentation 1 - left. Segmentation of fascicles in 3D from caudal (bottom/left) to cranial (top/right) for one left vagus nerve to the point at which most fascicles have merged (white) approximately 4 cm from all three branches entering the vagus nerve (excluding superior cardiac branches). The CS of the nerve at specified intervals from cervical level are displayed on the left. The cardiac fascicle present in the first CS is the superior cardiac fascicle(s). In the second segmentation figure from the left, after the recurrent laryngeal and cardiac fascicles enter the vagus nerve from their branches (right), the cardiac branches merge with the pulmonary fascicles shortly thereafter (left, pink). Segments shown on right are 4 cm in length.

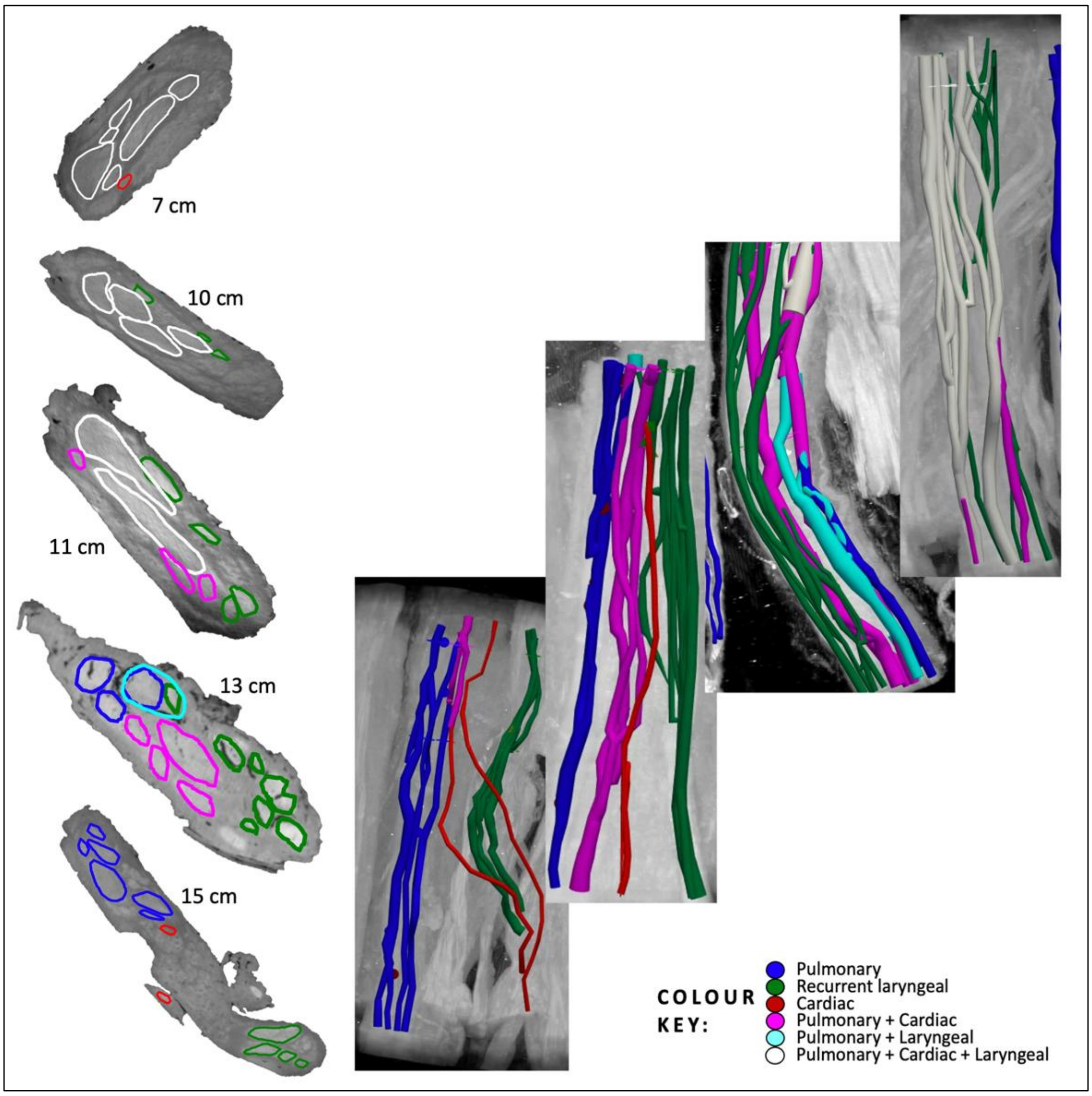

Supplementary Figure 3. Human vagus nerve segmentation 2 - left. Segmentation of fascicles in 3D from caudal (bottom/left) to cranial (top/right) for a second left vagus nerve to the point at which most fascicles have merged (white) approximately 4 cm from all three branches entering the vagus nerve (excluding superior cardiac branches). The CS of the nerve at specified intervals from cervical level are displayed on the left. The cardiac fascicle present in the first CS is the superior cardiac fascicle(s). Segments shown on right are 4 cm in length.

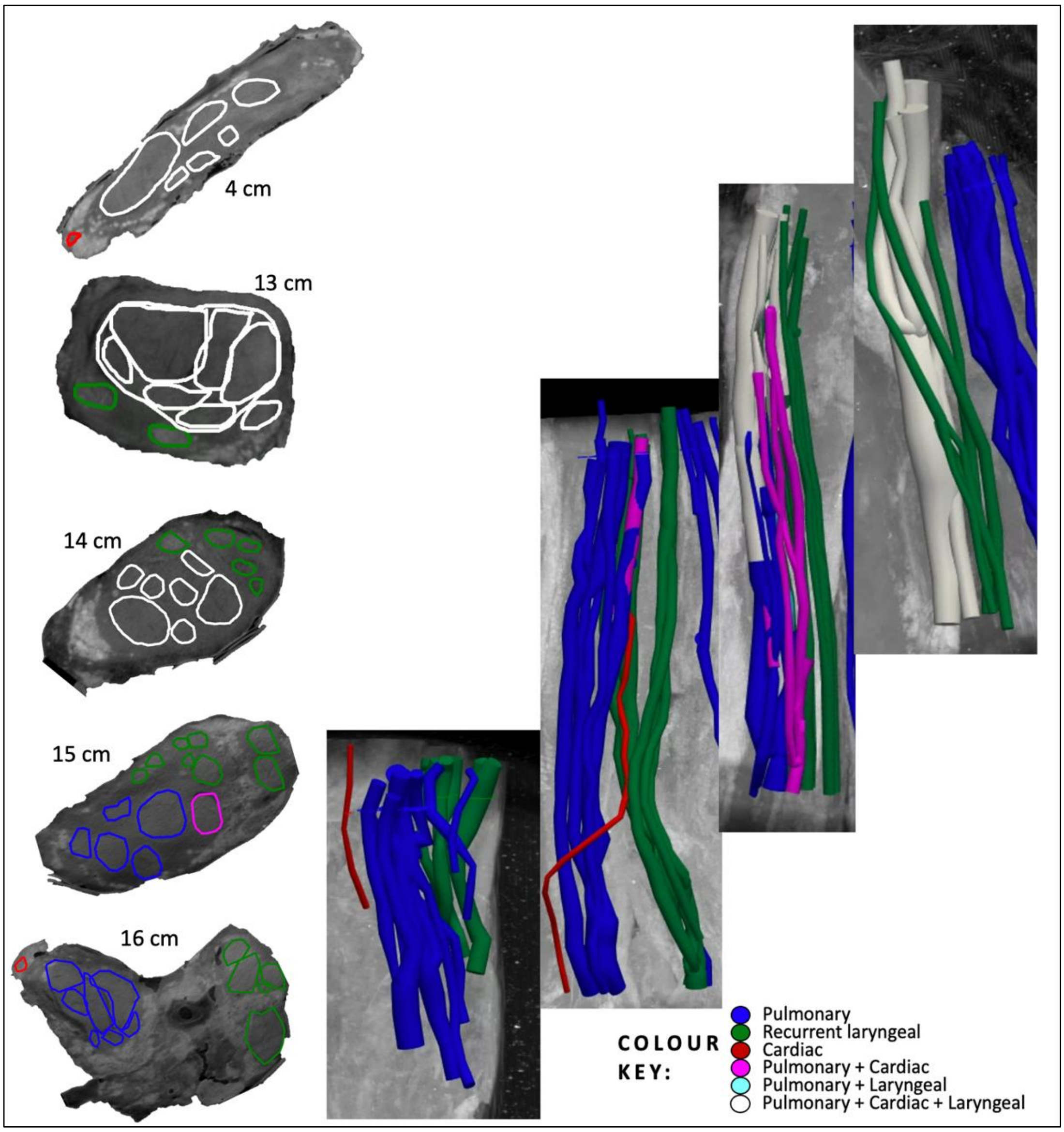

Supplementary Figure 4. Human vagus nerve segmentation 3 - left. Segmentation of fascicles in 3D from caudal (bottom/left) to cranial (top/right) for a third left vagus nerve to the point at which most fascicles have merged (white) approximately 4 cm from all three branches entering the vagus nerve (excluding superior cardiac branches). The CS of the nerve at specified intervals from cervical level are displayed on the left. The cardiac fascicle present in the first CS is the superior cardiac fascicle(s). Segments shown on right are 4 cm in length.

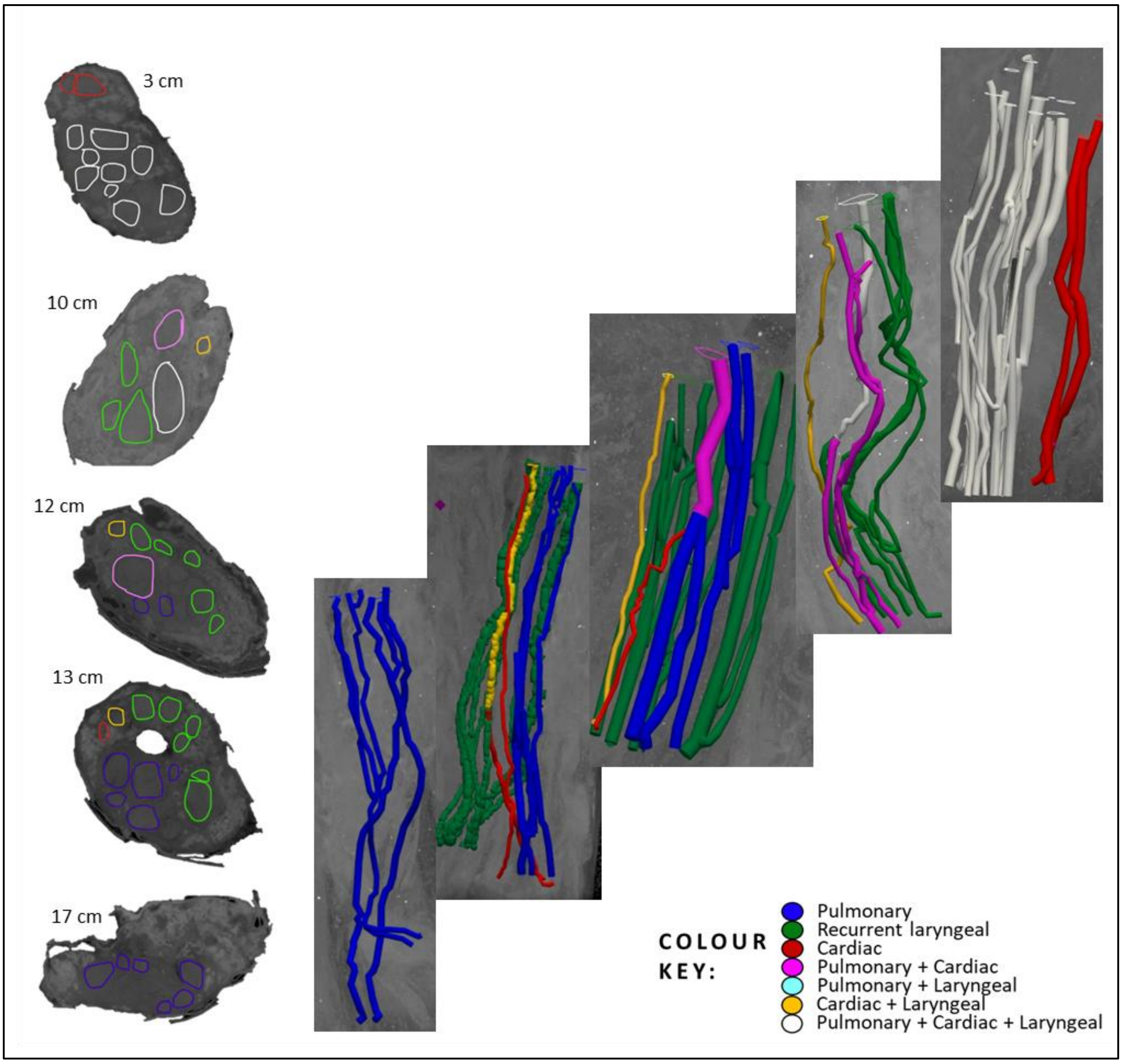

Supplementary Figure 5. Human vagus nerve segmentation 4 - right. Segmentation of fascicles in 3D from caudal (bottom/left) to cranial (top/right) for a fourth nerve from the right side, to the point at which most fascicles have merged (white) approximately 4 cm from all three branches entering the vagus nerve (excluding superior cardiac branches). The CS of the nerve at specified intervals from cervical level are displayed on the left. The cardiac fascicle present in the first CS is the superior cardiac fascicle(s). Segments shown on right are 4 cm in length.

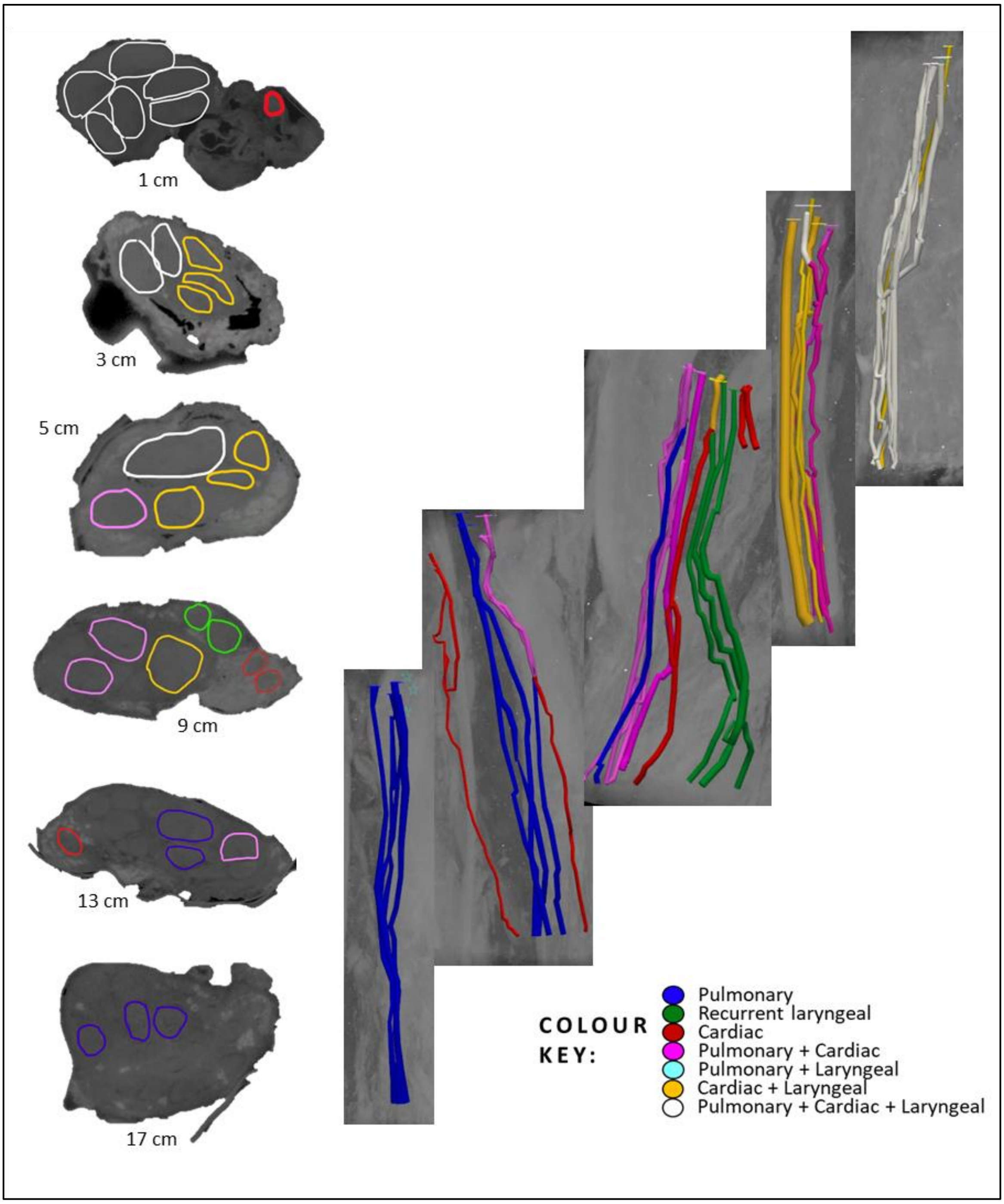

Supplementary Figure 6. Human vagus nerve segmentation 5 - right. Segmentation of fascicles in 3D from caudal (bottom/left) to cranial (top/right) for a fifth, right vagus nerve to the point at which most fascicles have merged (white) or fascicles containing cardiac and laryngeal fibers (yellow) approximately 4 cm from all three branches entering the vagus nerve (excluding superior cardiac branches). The CS of the nerve at specified intervals from cervical level are displayed on the left. The cardiac fascicle present in the first CS is the superior cardiac fascicle(s). Segments shown on right are 4 cm in length.

Supplementary Video 1. Example of anastomoses (splitting and merging) of fascicles in the human vagus nerve

https://youtube.com/shorts/0GgmLg2mN-0?feature=share

Supplementary Table 2. Repeatability assessment for morphometric measurements of the human vagus nerves

Researcher	*Repeated Measure*	*Human Nerve*	*Side*	*Circumfer-ence (mm)*	*Area (mm2)*	*Short Diameter (mm)*	*Long Diameter (mm)*	*Mean Diameter*
	1	*1*	*R*	9.12	3.68	1.04	3.73	2.39
	2	*1*	*R*	9.04	3.69	1.06	3.74	2.40
	3	*1*	*R*	9.08	3.72	1.01	3.77	2.39
	4	*1*	*R*	9.03	3.71	1.02	3.74	2.38
1	*5*	*1*	*R*	9.04	3.65	1.04	3.74	2.39
	6	*1*	*R*	9.05	3.70	1.04	3.75	2.40
	7	*1*	*R*	9.00	3.70	1.03	3.73	2.38
	8	*1*	*R*	9.06	3.69	1.02	3.77	2.40
	9	*1*	*R*	9.16	3.62	1.03	3.74	2.39
			Mean	9.06	3.68	1.03	3.75	2.39
			Std Dev	0.05	0.03	0.01	0.01	0.01
			Variance	0.00	0.00	0.00	0.00	0.00
			95% CI	0.03	0.02	0.01	0.01	0.00
	1	*1*	*R*	9.12	3.69	1.03	3.74	2.39
	2	*1*	*R*	9.06	3.70	1.04	3.75	2.40
	3	*1*	*R*	9.10	3.71	1.03	3.76	2.40
	4	*1*	*R*	9.08	3.68	1.03	3.74	2.39
2	*5*	*1*	*R*	9.04	3.72	1.05	3.74	2.40
	6	*1*	*R*	9.07	3.71	1.03	3.76	2.40
	7	*1*	*R*	9.10	3.68	1.04	3.77	2.41
	8	*1*	*R*	9.07	3.69	1.03	3.75	2.39
	9	*1*	*R*	8.99	3.66	1.05	3.76	2.41
		Intra-observer	***Mean***	9.07	3.69	1.04	3.75	2.39
			Std Dev	0.04	0.02	0.01	0.01	0.01
			Variance	0.00	0.00	0.00	0.00	0.00
			95% CI	0.02	0.01	0.01	0.01	0.00
	1	*1*	*R*	9.13	3.71	1.02	3.73	2.38
	2	*1*	*R*	9.09	3.68	1.05	3.74	2.40
	3	*1*	*R*	9.10	3.71	1.04	3.74	2.39
	4	*1*	*R*	9.11	3.74	1.04	3.73	2.39
3	*5*	*1*	*R*	9.06	3.68	1.03	3.75	2.39
	6	*1*	*R*	9.04	3.68	1.03	3.75	2.39
	7	*1*	*R*	9.10	3.73	1.03	3.76	2.40
	8	*1*	*R*	9.09	3.71	1.05	3.75	2.40
	9	*1*	*R*	9.08	3.73	1.04	3.75	2.40
			Mean	9.09	3.71	1.04	3.74	2.39
			Std Dev	0.03	0.02	0.01	0.01	0.01

	Variance	0.00	0.00	0.00	0.00	0.00
	95% CI	0.02	0.01	0.01	0.01	0.00
		p-values for inter-observer comparisons (Tukey-corrected pairwise tests following one-way ANOVA)				
	Researchers 1 - 2	0.95	0.69	0.69	0.49	0.25
	Researchers 2 - 3	0.40	0.13	0.69	0.98	0.88
	Researchers 1 - 3	0.57	0.47	1.00	0.38	0.49

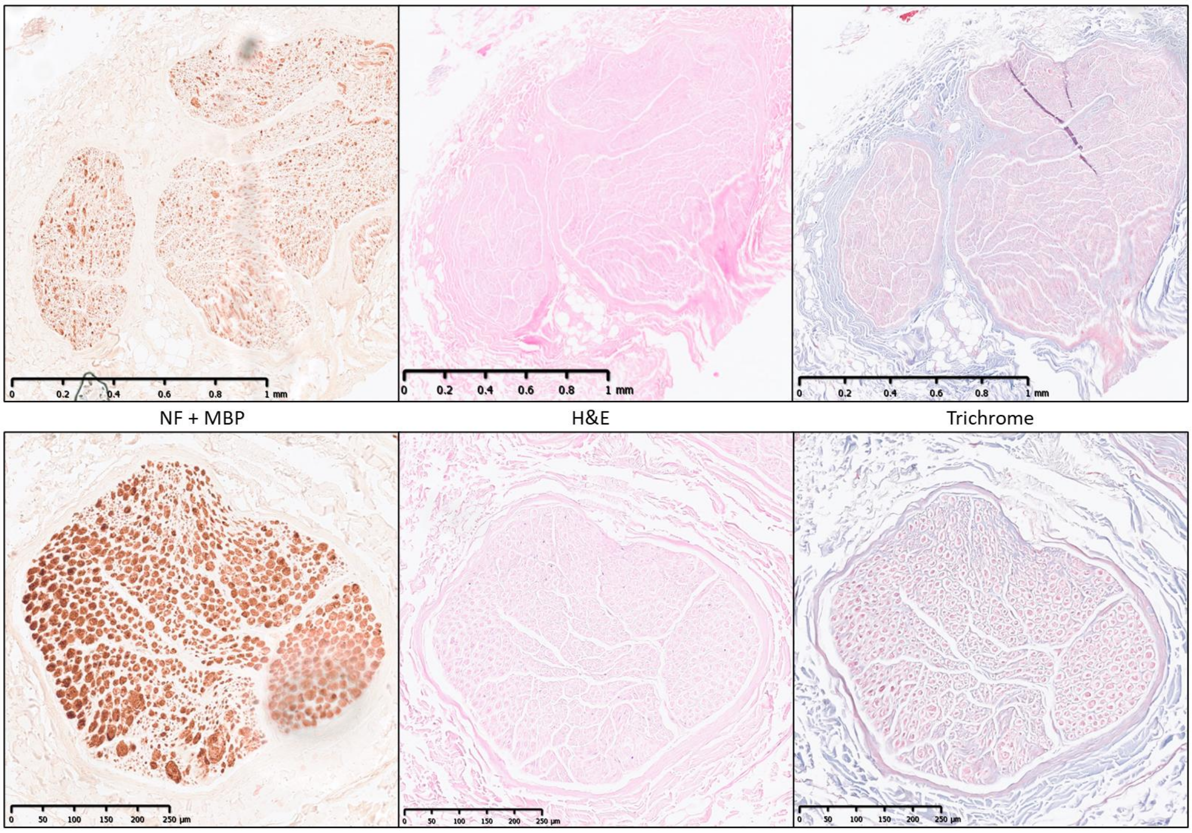

Supplementary Figure 7. Two examples of the different histological and immunohistochemical stains used. Cross-sections from two nerves with each of the histological or immunohistochemical stains including double staining with neurofilament (NF) and myelin basic protein (MBP), hematoxylin and eosin (H&E), and Trichome staining, from left to right, respectively. This was performed for all 10 nerves at numerous intervals along the nerve.

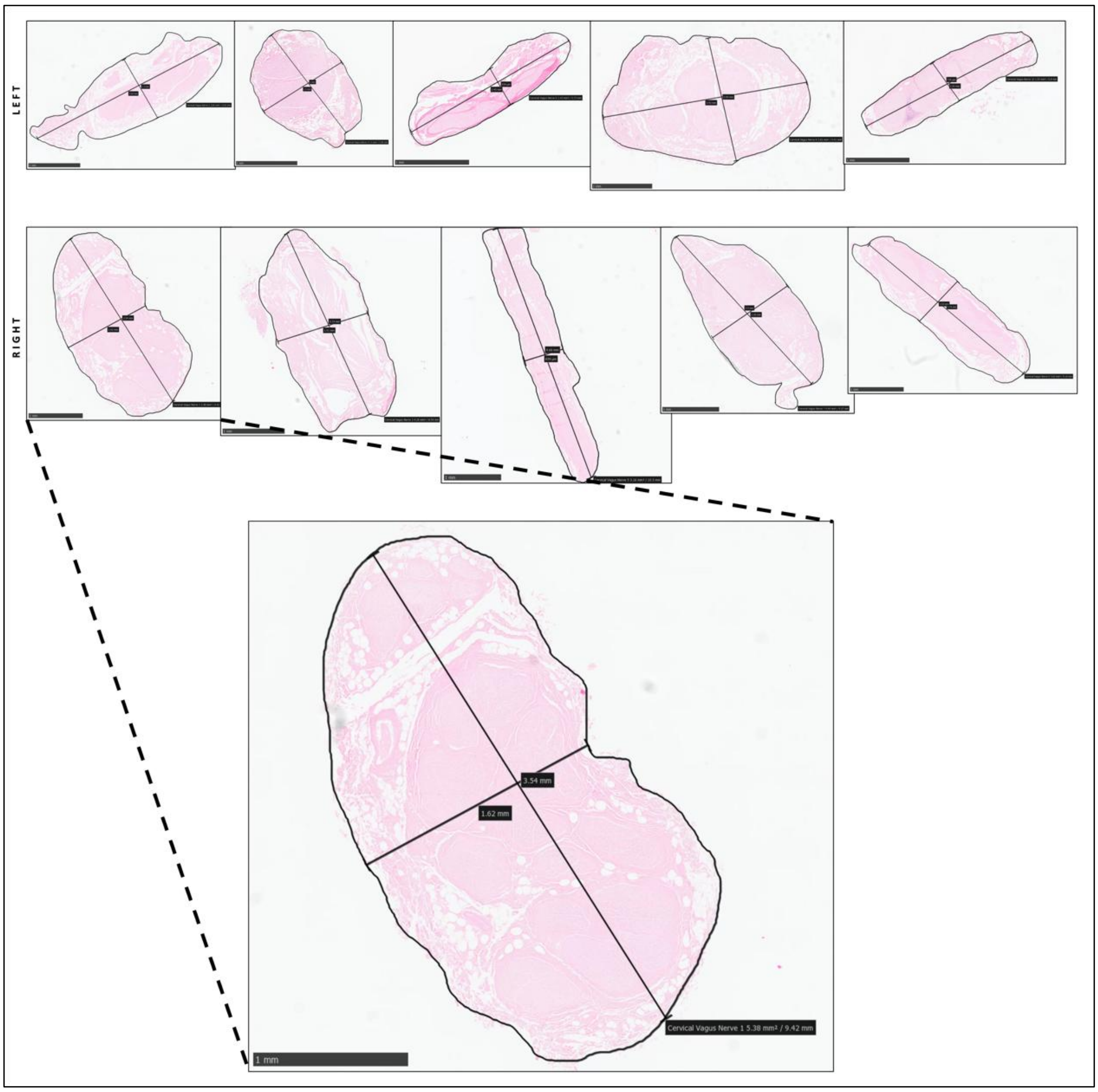

Supplementary Figure 8. Histology cross section measurements. Cross sections from mid-cervical level of five left and five right human vagus nerves (n=10) stained with H&E displaying two diameter (short and long) and circumference measurements for each for morphological analysis.

Supplementary Table 3. Morphological analysis of the human cervical vagus nerve (n=10)

Human Nerve	*Side*	*Circumfer-ence (mm)*	*Area (mm2)*	*Short Diameter (mm)*	*Long Diameter (mm)*	*Mean Diameter*	*No. of fascicles*	*No. of fascicle bundles*
1	R	9.42	5.38	1.62	3.52	2.57	22.00	13.00
2	L	9.92	3.82	1.30	3.94	2.62	14.00	5.00
3	R	8.59	4.26	1.51	3.19	2.35	7.00	4.00
4	L	5.89	2.40	1.50	2.00	1.75	6.00	4.00
5	R	10.50	3.16	0.70	4.66	2.68	12.00	4.00
6	L	5.75	1.43	0.54	2.45	1.50	9.00	3.00
7	R	9.37	4.44	1.59	3.50	2.55	11.00	7.00
8	L	9.41	5.82	2.09	3.43	2.76	11.00	6.00
9	R	9.16	3.62	1.03	3.74	2.39	8.00	8.00
10	L	6.80	1.93	0.73	2.93	1.83	8.00	7.00
Mean		8.48	3.63	1.26	3.34	2.30	10.80	6.10
Std Dev		1.70	1.43	0.50	0.75	0.44	4.64	2.92
Variance		2.62	1.84	0.22	0.51	0.18	19.36	7.69
CI (95%)		1.22	1.02	0.35	0.54	0.32	3.32	2.09
Mean L		7.55	3.08	1.23	2.95	2.09	9.60	5.00
Std Dev L		1.98	1.77	0.62	0.77	0.56	3.05	1.58
Variance L		3.13	2.51	0.31	0.47	0.25	7.44	2.00
CI L (95%)		2.45	2.20	0.77	0.95	0.70	3.79	1.96
Mean R		9.41	4.17	1.29	3.72	2.51	12.00	7.20
Std Dev R		0.69	0.85	0.41	0.56	0.14	5.96	3.70
Variance R		0.38	0.57	0.13	0.25	0.01	28.40	10.96
CI R (95%)		0.86	1.05	0.51	0.70	0.17	7.40	4.60

Supplementary Table 4. Fascicle and fascicle bundle counts from regular intervals of the vagus nerve trunk and organ-specific branches

Row 1 – Fascicles	*1cm*	*3cm*	*5cm*	*9cm*	*13cm*	*17cm*	*21cm*	*Mean*	*Std Dev*
Row 2 – Bundles									
Row 3 – Fascicles	*Superior cardiac*	*Recurrent laryngeal*	*Inferior cardiac*	*Pulmonary*					
Row 4 – Bundles									
Nerve 1	11	22	20	18	24	15	N/A	18.00	4.52
	9	13	17	12	12	10	N/A	11.83	2.79
	4	/	3	3					
	3	/	3	3					
Nerve 2	8	14	14	22	12	N/A	N/A	14.00	5.10
	6	5	10	15	11	N/A	N/A	9.40	4.04
	2	7	2	/					
	2	6	2	/					
Nerve 3	7	6	8	9	21	13	N/A	10.67	5.61
	4	5	4	8	12	8	N/A	6.83	3.13
	3	/	2	5					
	3	/	2	5					
Nerve 4	6	4	7	31	7	7	N/A	10.33	10.19
	4	2	3	14	6	6	N/A	5.83	4.31
	6	8	4	6					
	6	8	4	4					
Nerve 5	15	12	11	12	17	6	8	11.57	3.78
	7	4	9	11	14	4	8	8.14	3.97
	2	10	1	7					
	2	10	1	7					
Nerve 6	9	11	9	16	1	7	N/A	8.83	4.92
	3	8	3	13	1	6	N/A	5.67	4.37
	1	8	2	6					
	1	7	2	6					
Nerve 7	11	11	20	9	16	14	N/A	13.50	4.04
	7	7	16	7	13	8	N/A	9.67	3.88
	4	5	3	4					
	4	4	3	3					
Nerve 8	3	4	11	23	14	7	N/A	10.33	7.47
	2	4	6	22	12	4	N/A	8.33	7.53
	4	7	1	/					
	4	6	1	/					
Nerve 9	8	9	12	11	28	8	25	14.43	8.42

	5	7	11	9	25	7	19	11.86	7.31
	2	4	3	12					
	2	4	3	8					
Nerve 10	5	6	8	11	9	1	8	6.86	3.24
	5	5	7	7	7	1	5	5.29	2.34
	3	9	/	/					
	3	4	/	/					
Mean	8.30	9.90	12.00	16.20	14.90	8.67	13.67	11.85	5.75
	5.20	6.00	8.60	11.80	11.30	6.00	10.67	8.29	4.37
	3.10	7.25	2.33	6.14					
	3.00	6.13	2.33	5.14					
Std Dev	3.43	5.49	4.71	7.28	8.14	4.50	9.81	3.20	2.24
	2.10	3.02	5.02	4.59	6.25	2.69	7.37	2.42	1.74
	1.45	1.98	1.00	2.91					
	1.41	2.17	1.00	1.95					

Supplementary Table 5. Trichrome cross sections at regular intervals along the trunk of the vagus and from organ-specific branches

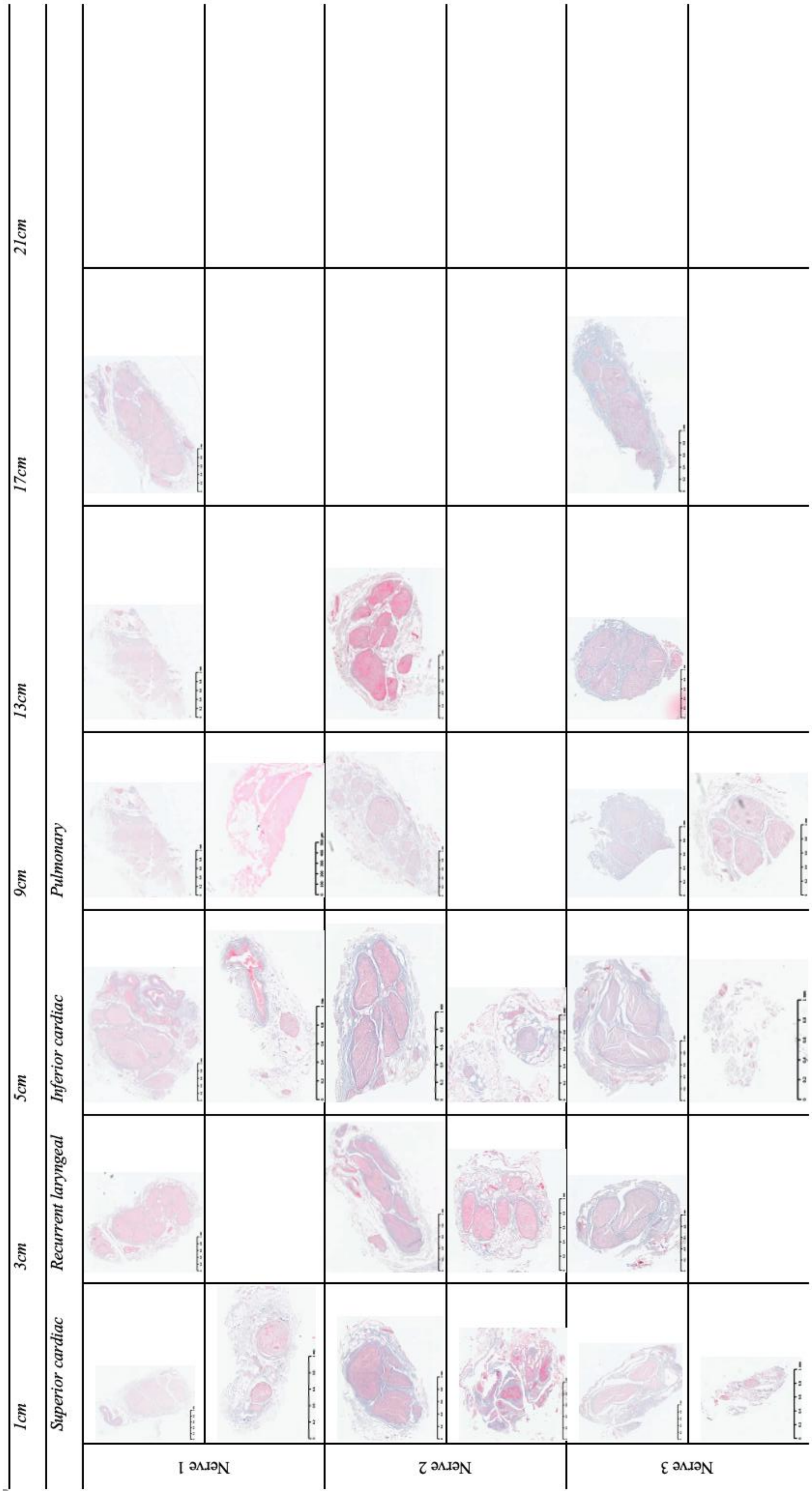

Nerve 7	Nerve 6	Nerve 5	Nerve 4

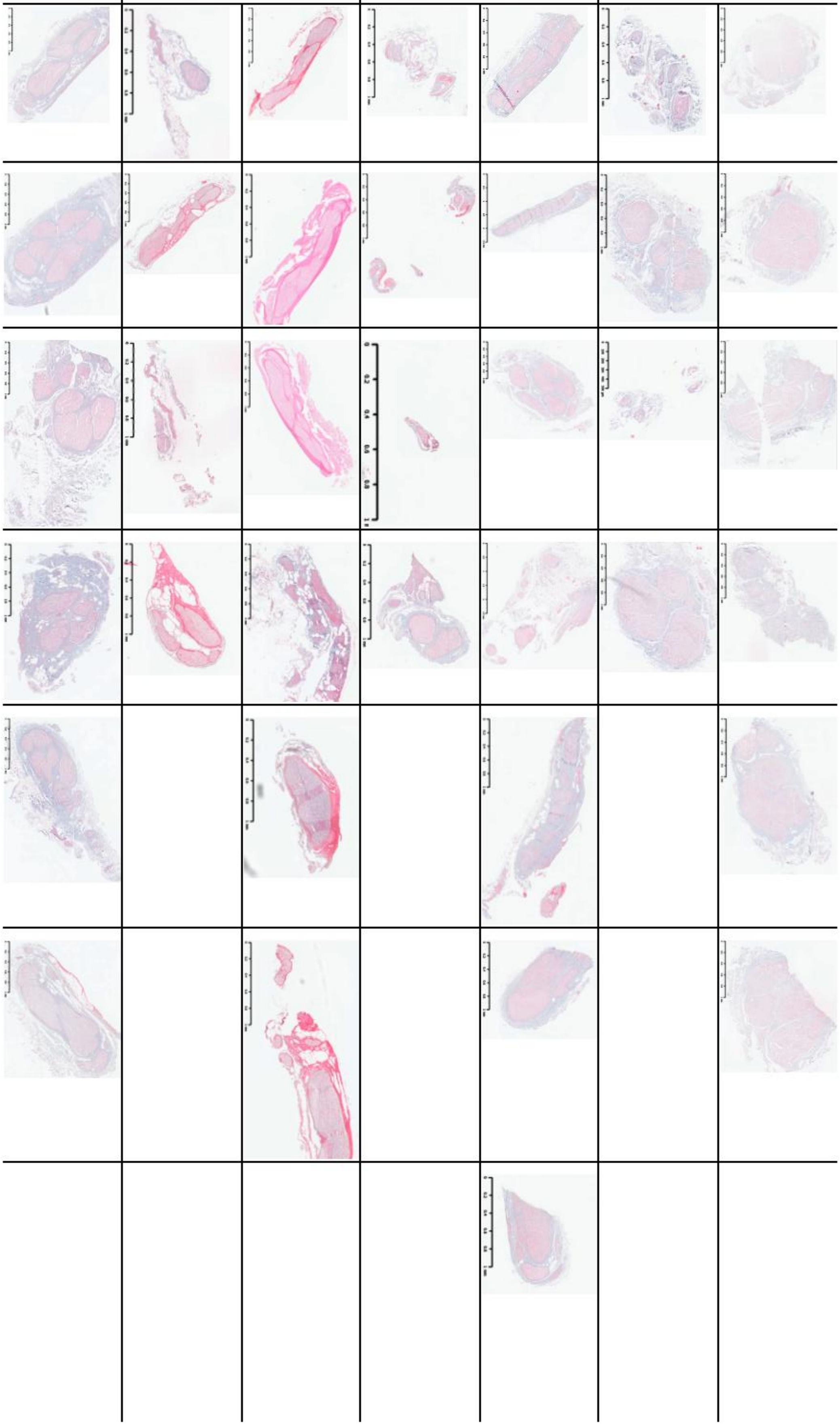

Nerve 10	Nerve 9	Nerve 8

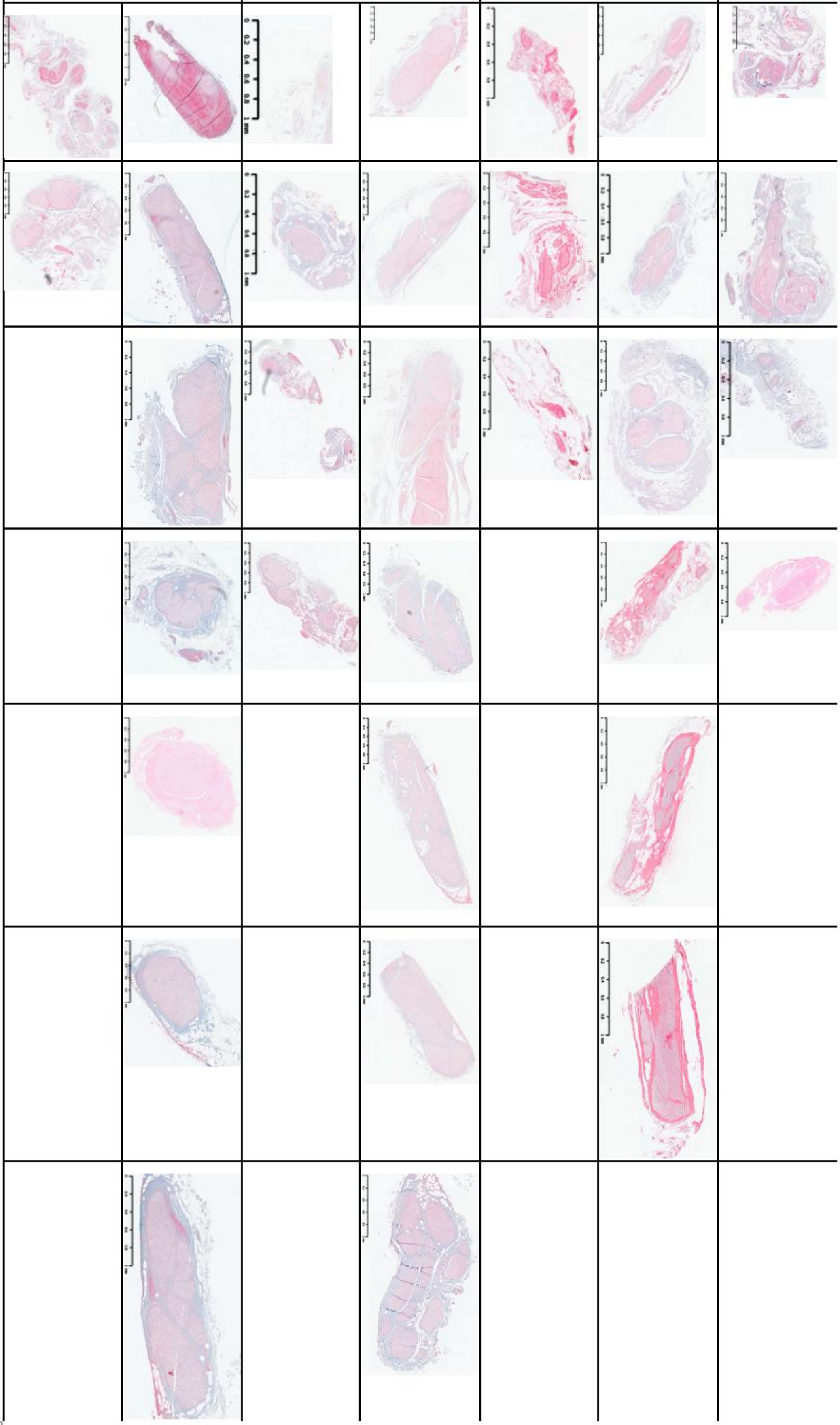

Supplementary Table 6. Human vagus nerve branch measurements: distances from cervical level to organ-specific branches and distance between branches exiting the vagus nerve

Distance from cervical level (cm)

Nerve	1	2	3	4	5	6	7	8	9	10
Side	R	L	R	L	R	L	R	L	R	L
Superior cardiac (SC)	4.5	3.75	8	3	7	7	10.5	8	8	4
Recurrent laryngeal (RL)	10.5	15	10	15	14	13.5	13.5	15.5	13.5	17.5
Inferior cardiac 1 (IC1)	10.5	14.5	10	15	14	13.5	13.5	15	13.5	17
Inferior cardiac 2 (IC2)	11	15	10.5	15.5	15	13.5	14	15.5	14	17.5
Pulmonary 1 (P1)	18	17	16.5	16	20	16	19	18	20.5	19
Pulmonary 2 (P2)	18.5	17.5	17	18	21	18	21	19	21	21
Pulmonary 3 (P3)	19	N/A	17.5	N/A	22	19	N/A	N/A	21.5	N/A

	Mean	***Std Dev***	***Variance***	***CI (95%)***	***Mean R***	***Std Dev R***	***Var R***	***CI R***	***Mean L***	***Std Dev L***	***Var L***	***CI L***
SC	6.38	2.43	5.32	1.74	7.60	2.16	3.74	2.68	5.15	2.21	3.89	2.74
RL	13.80	2.24	4.51	1.60	12.30	1.89	2.86	2.35	15.30	1.44	1.66	1.79
IC1	13.65	2.08	3.90	1.49	12.30	1.89	2.86	2.35	15.00	1.27	1.30	1.58
IC2	14.15	2.11	4.40	1.59	12.90	2.01	3.24	2.50	15.40	1.43	0.92	1.38
P1	18.00	1.62	2.35	1.16	18.80	1.60	2.06	1.99	17.20	1.30	1.36	1.62
P2	19.20	1.64	2.41	1.17	19.70	1.86	2.76	2.31	18.70	1.40	1.56	1.73
P3	19.80	1.89	2.86	1.35	20.00	2.12	3.38	2.63	19.00	N/A	N/A	N/A

Distance between branches (cm)

Nerve	1	2	3	4	5	6	7	8	9	10
Side	R	L	R	L	R	L	R	L	R	L
SC - RL	6	11.25	2	12	7	6.5	3	7.5	5.5	13.5
RL - IC1	0	-0.5	0	0	0	0	0	-0.5	0	-0.5
IC1 - IC2	0.5	0.5	0.5	0.5	1	0	0.5	0.5	0.5	0.5
RL - P1	7.5	2	6.5	1	6	2.5	5.5	2.5	7	1.5
IC2 - P1	7	2	6	0.5	5	2.5	5	2.5	6.5	1.5
P1 - P2	0.5	0.5	0.5	2	1	2	2	1	0.5	2
P2 - P3	0.5	N/A	0.5	N/A	1	1	N/A	N/A	0.5	N/A

	Mean	***Std Dev***	***Mean R***	***Std Dev R***	***Mean L***	***Std Dev L***
SC - RL	7.43	3.78	4.70	2.11	10.15	3.01
RL - IC1	-0.15	0.24	0.00	0.00	-0.30	0.27
IC1 - IC2	0.50	0.24	0.60	0.22	0.40	0.22
RL - P1	4.20	2.52	6.50	0.79	1.90	0.65
IC2 - P1	3.85	2.31	5.90	0.89	1.80	0.84
P1 - P2	1.20	0.71	0.90	0.65	1.50	0.71
P2 - P3	0.70	0.27	0.63	0.25	1.00	N/A
